# Intratumoral synthesis of transformable metal-phenolic nanoaggregates with enhanced tumor penetration and retention for photothermal immunotherapy

**DOI:** 10.7150/thno.74808

**Published:** 2022-08-29

**Authors:** Xianglian He, Hongfu Zhu, Jiaojiao Shang, Meifeng Li, Yaoyao Zhang, Shicheng Zhou, Guidong Gong, Yunxiang He, Anna Blocki, Junling Guo

**Affiliations:** 1 BMI Center for Biomass Materials and Nanointerfaces, College of Biomass Science and Engineering, Sichuan University, Chengdu, Sichuan 610065, China.; 2 Collage of Material Science and Engineering, Sichuan University, Chengdu, Sichuan 610065, China.; 3 Department of Biomass Chemistry and Engineering, College of Biomass Science and Engineering, Sichuan University, Chengdu, Sichuan 610065, China.; 4 Key Laboratory of Birth Defects and Related of Women and Children of Ministry of Education, Department of Pediatrics, The Reproductive Medical Center, Department of Obstetrics and Gynecology, West China Second University Hospital, Sichuan University, Chengdu, Sichuan 610041, China.; 5 Department of Bioengineering, School of Engineering, The University of Tokyo, 7-3-1 Hongo, Bunkyo-ku, Tokyo 113-8656, Japan.; 6 Institute for Tissue Engineering and Regenerative Medicine, The Chinese University of Hong Kong (CUHK), Shatin, Hong Kong SAR, China.; 7 State Key Laboratory of Polymer Materials Engineering, Sichuan University, Chengdu, Sichuan 610065, China.; 8 Bioproducts Institute, Department of Chemical and Biological Engineering, University of British Columbia, Vancouver, BC V6T 1Z4, Canada.

**Keywords:** metal-phenolic coordination, intratumoral self-assembly, pseudo-stepwise kinetics, transformable materials, photothermal immunotherapy

## Abstract

**Rationale:** Effective photothermal therapy (PTT) remains a great challenge due to the difficulties of delivering photothermal agents with both deep penetration and prolonged retention at tumor lesion spatiotemporally.

**Methods**: Here, we report an intratumoral self-assembled nanostructured aggregate named FerH, composed of a natural polyphenol and a commercial iron supplement. FerH assemblies possess size-increasing dynamic kinetics as a pseudo-stepwise polymerization from discrete nanocomplexes to microscale aggregates.

**Results**: The nanocomplex can penetrate deeply into solid tumors, followed by prolonged retention (> 6 days) due to the *in vivo* growth into nanoaggregates in the tumor microenvironment. FerH performs a targeting ablation of tumors with a high photothermal conversion efficiency (60.2%). Importantly, an enhanced immunotherapeutic effect on the distant tumor can be triggered when co-administrated with checkpoint-blockade PD-L1 antibody.

**Conclusions**: Such a therapeutic approach by intratumoral synthesis of metal-phenolic nanoaggregates can be instructive to address the challenges associated with malignant tumors.

## Introduction

Photothermal therapy (PTT) performs as a non-invasive and precise approach to cancer treatment 1. During the PTT, hyperthermia generated by photothermal agents (PTAs) administration and near-infrared (NIR, 700-900 nm) irradiation causes irreversible damage to the tumor tissue to rapidly ablate tumor cells 2,3. Meanwhile, PTT can also trigger anti-tumor immunity by inducing immunogenetic cell death 4,5. Particularly, deep penetration and long retention of PTAs at tumor lesions are two key criteria for effective PTT 3,6,7. Molecular and nanoscale PTAs (< 100 nm), obtained through delicate synthesis and controlled self-assembly, can penetrate through the entire tumor 6,8,9. However, the nanosystems cannot escape from the barrier of metabolism leading to a rapid clearance from the bloodstream within a few hours, which results in an insufficient accumulation and the corresponding suboptimal PTT efficiency 10,11. Despite being more advantageous for retention time prolongation, the large-sized PTAs (> 100 nm) show the difficulties of deep penetration, heavily impeding the complete ablation of complete or relatively large-sized (> 500 mm^3^) tumors 12. Therefore, it remains a great challenge to deliver PTAs with deep penetration and prolonged retention concurrently.

*In situ* self-assembly or morphological transformation of nanosystems triggered by the tumor microenvironment (TME) has been demonstrated as an efficient strategy to design smart therapeutic agents in tumor tissues 13,14. Peptides or peptide-polymer conjugates modified with biologically-responsive moieties can penetrate deeply into the tumor tissues. When stimulated by the receptor proteins 15, enzymes 16, or temperature changes 17, the stimuli-responsiveness induced assembly initiates the intercellular accumulation in the tumor tissues. Therefore, it can be rationalized that *in situ* and *in vivo* synthesis of nanosystems provides an opportunity to combine the merits of multiscale PTAs, which ascribes to the size-increasing property and aggregation into certain topological structures of the tumor to achieve both sufficient penetration and retention spatiotemporally.

**Figure 1:**
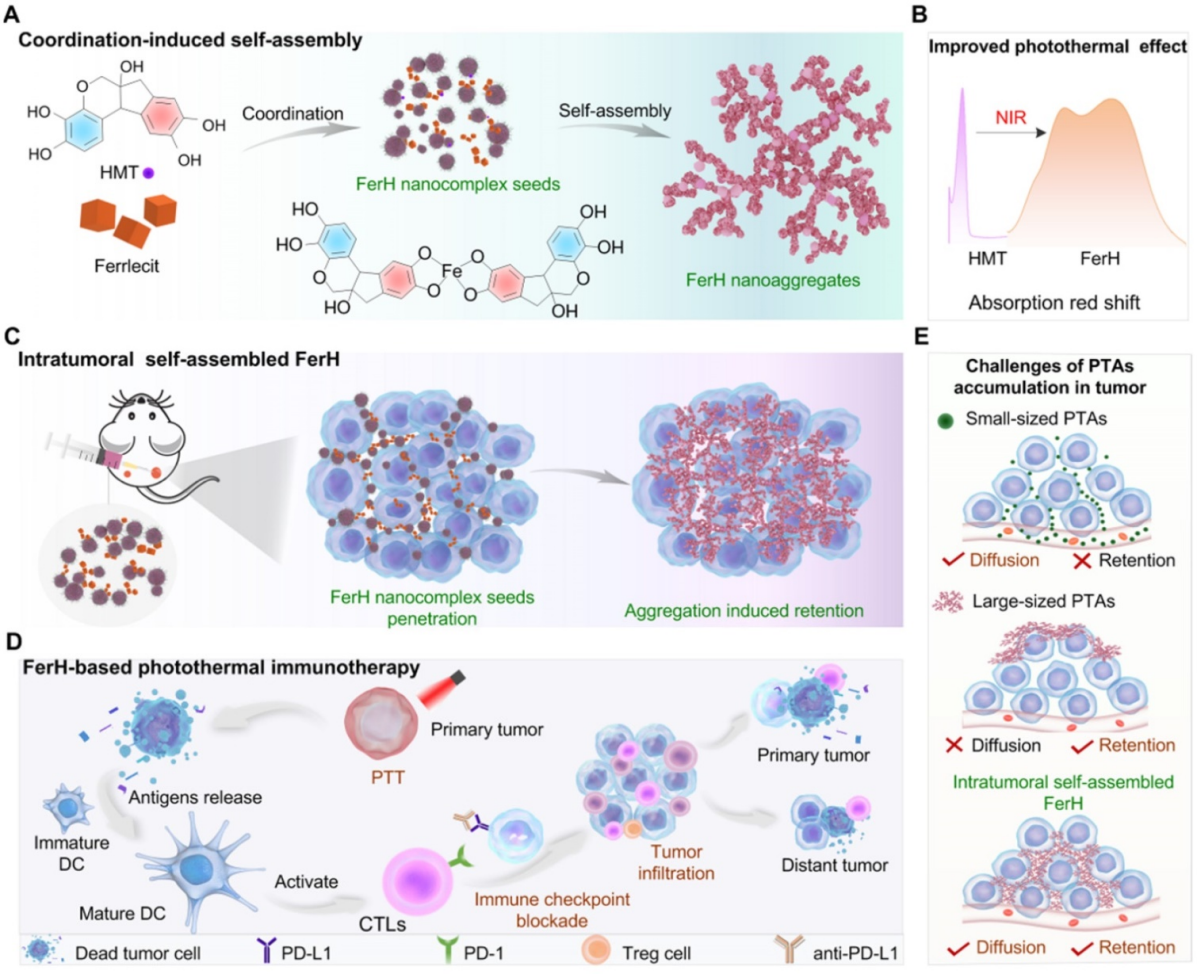
** Schematic illustration of HMT and Fe^3+^ ion coordination induced FerH self-assembly for photothermal immunotherapy. (A)** Formation of FerH nanocomplex seeds and FerH nanoaggregates. **(B)** Bathochromic shift on the UV-Vis spectra of FerH complex attributed to the Fe^3+^ ions and HMT coordination, endowing effective photothermal property. **(C)** Schematic of intratumoral self-assembly of FerH nanoaggregates with interconnected nanostructure *in vivo*. **(D)** FerH co-administrated with PD-L1 checkpoint blockade for immune response strengthening to inhibit tumor proliferation. **(E)** Intratumoral synthesis of FerH nanoaggregates overcomes the challenges of conventional PTAs.

Natural polyphenol, a plant-based bio-building block, provides eligible solutions to the concurrent behavior of PTAs in terms of *in situ* stimuli-responsive self-assembly. Polyphenols can coordinate with metal ions rapidly to form metal-phenolic network (MPN) complexes, after which assemble into network-like aggregates or higher-level assemblies 18-20. The facile self-assembly of MPN complexes has been demonstrated to be tailored in a controlled manner to enable diverse morphologies and functionalities 21-24, which has been greatly explored in many biomedical applications due to their biocompatibility, pH-responsiveness, and modular compositions 25-37. Here, we report an *in vivo* stimuli-responsive assembled PTA constructed from a natural polyphenol (hematoxylin from *Haematoxylum campechianum*, HMT) and an iron supplement Ferrlecit^TM^ in the tumor tissue for PTT (**Figure 1**). Specifically, HMT coordinates with the ferric ions (Fe^3+^) from Ferrlecit^TM^ to form FerH nanocomplex seeds, allowing for a deep tumor penetration. The penetrated FerH nanocomplex seeds possess a dynamic size-growing property based on the stepwise coordination to form interconnected nanoaggregates in the tumor, thus endowing a prolonged retention time (**Figure 1A, C**). The obtained FerH nanoaggregates exhibit a recorded-high photothermal conversion efficiency in the NIR region compared with previously reported metal-phenolic materials (**Figure 1B**). Importantly, FerH performed an effective eradication of the primary tumor with a single-dose administration and successive laser irradiation. Moreover, FerH-mediated PTT triggers immunogenicity when coupled with the use of a programmed-cell-death ligand-1 checkpoint-blockade agent (*i.e.* anti-PD-L1), exhibiting a remarkable inhibition of distant tumors (**Figure 1D**). Collectively, this *in situ* and *in vivo* synthesis of MPN-based PTA provides a promising strategy for the next-generation PTT by overcoming the synthetic challenges of *in vivo* synthesis of multiscale materials for deep penetration and prolonged retention in solid tumors concurrently (**Figure 1E**).

## Preparation and characterization of FerH

**Figure 2:**
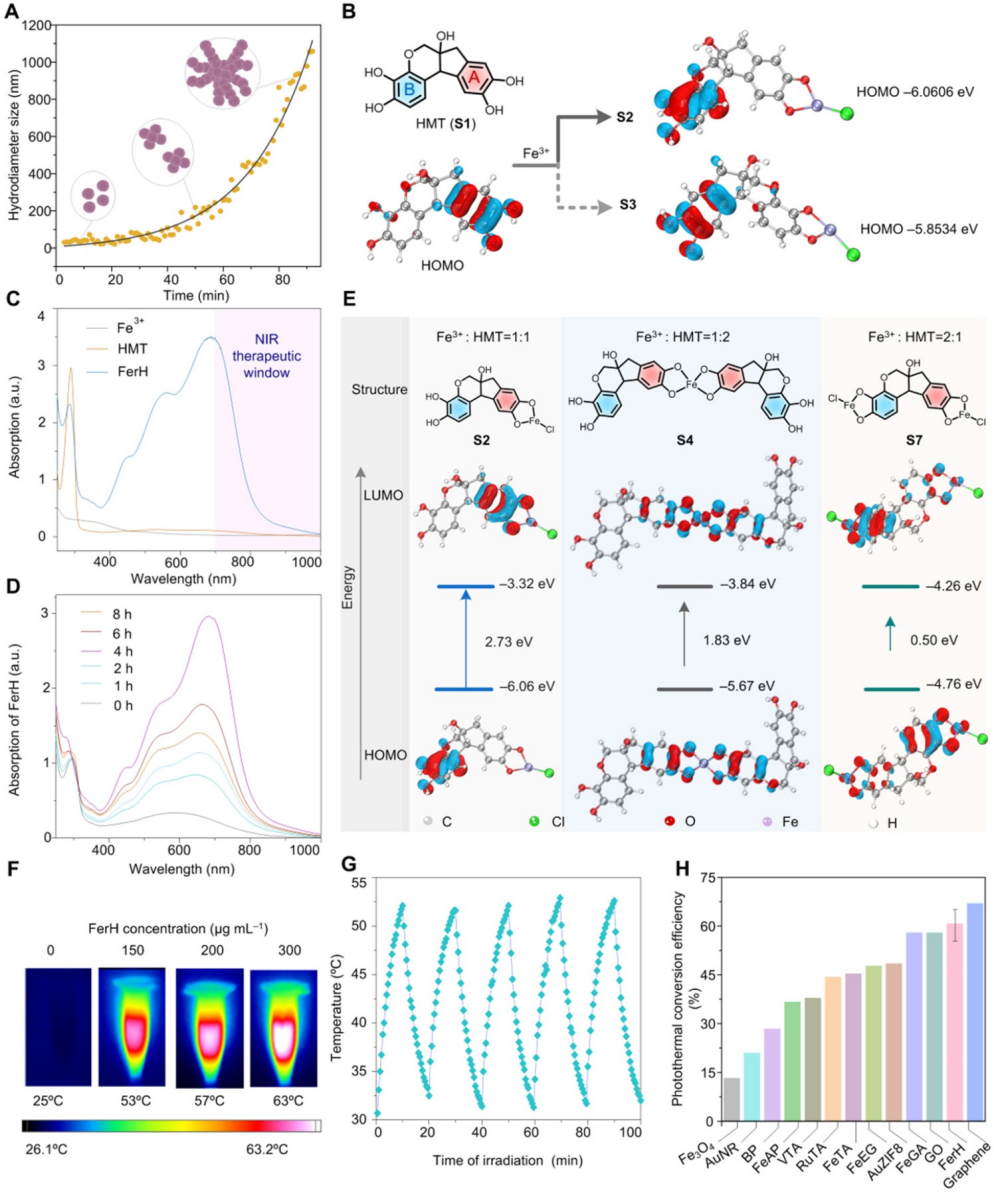
** Characterization, self-assembly process, and photothermal property of FerH. (A)** The increase of hydrodiameter size of FerH during the self-assembly. **(B)** Different electron densities of Ring A and B in HMT and preferred formation of coordination compound **S2** over **S3** after adding Fe^3+^ ions. **(C)** UV-Vis-NIR absorption spectrum of HMT, Fe^3+^ ions, and FerH. Strong absorption in the NIR region was observed by FerH. **(D)** UV-Vis-NIR absorption spectra of FerH as a function of time. **(E)** Calculation of the HOMO-LUMO gaps of FerH complexes at Fe^3+^ ion to HMT ratios at 1:1 (**S2**), 1:2 (**S4**), and 2:1 (**S7**). The red and cyan colors denote positive and negative phases of orbitals whose isovalues are equal to 0.05. **(F)** The photothermal effect of FerH at different concentrations (150, 200, 300 μg mL^‒1^) after being irradiated by 808 nm laser (1 W cm^‒2^) for 10 min. **(G)** Photostability of FerH (150 μg mL^‒1^) by photothermal heating (1 W cm^‒2^) and natural cooling for 5 cycles. **(H)** Comparison of photothermal conversion efficiency of FerH and reported other photothermal agents.

A coordination-induced self-assembly method was employed to synthesize FerH nanoaggregates with the molar ratio of HMT and Fe^3+^ ions at 1:1 (see Methods for details). The solution changed from light brown to dark blue over time, suggesting the coordination between HMT and Fe^3+^ ion (**Figure S1**). A trend of the FerH size increase was observed by transmission electron microscopy (TEM) from discrete nanocomplex seeds in the light brown solution to the large nanoaggregates in the dark blue solution, respectively (**Figure S2**), which was consistent with the exponential growth curve observed by dynamic light scattering (DLS) (**Figure 2A**). The curve showed that the growth kinetics of FerH nanoaggregates was similar to that of the stepwise polymerization (**Figure S3**). The curve was further fitted with exponential functions to extract the rate information (see Data Fitting for details). A further linear fitting of ln(D) vs. *t* demonstrated a first-order model for the FerH growth kinetics, suggesting the dependence of the cluster size on the concentration of the FerH nanocomplex seeds (**Figure S4**). The growth curves of FerH prepared at the different molar ratios (2:1 and 1:2) of HMT and Fe^3+^ ions were investigated, indicating that the molar ratio of HMT and Fe^3+^ ions have a negligible influence on the lag time of FerH (**Figure S5**).

Computational studies were subsequently employed to study the stepwise size-growing mechanism based on the coordination between HMT and Fe^3+^ ion. Regarding the molar ratio at 1:1, several structures of coordination compounds were proposed as subunits for nanocomplex seeds (**Figure S6**). The asymmetric HMT (**S1**) contains two catechol groups (Ring A and B), which coordinate with the Fe^3+^ ions at different rates. The optimized structure of HMT showed different electron densities on two aromatic rings with Ring A possessing more electrons, which suggests a higher activity of Ring A for the coordination with Fe^3+^ ions. As a result, most Fe^3+^ ions prefer to coordinate with Ring A to form B-HMT-A-Fe^3+^ (**S2**) over A-HMT-B-Fe^3+^ (**S3**). The calculated energy of HOMO orbitals showed a lower value for **S2** (-6.06 eV) compared with **S3** (-5.85 eV) (**Figure 2B**). The successive coordination of **S2** with excessive **S1** or **S2** itself would allow for the formation of complexes (**S4** and **S5**) containing two HMT ligands and one Fe^3+^ ion. **S6** would be less favored due to the slow kinetics of Ring B. Chances are also there for the formation of complexes containing one HMT ligand and two Fe^3+^ ions (**S7**). Electrospray ionization mass spectrometry (ESI-MS) demonstrated the presence of the mentioned three key coordination compounds with the m/z peaks found as 390.9 for 1:1, 480.8 for 1:2, and 762.9 for 2:1 (**Figure S7**). As the concentration of different HMT-Fe^3+^ complexes increases, coordination-induced self-assembly occurs to form nanocomplexes, and further form nanoaggregates rapidly. The formation of FerH nanoaggregates in stages contributes to the appearance of the lag period in the size growth curve.

The coordination of HMT and Fe^3+^ ions was further characterized by Fourier transform infrared (FTIR) spectroscopy with the reduced stretching vibration of O‒H bonds in the high frequency region (**Figure S8**). Raman spectroscopy (**Figure S9**) confirmed the coordination with a Fe‒O vibration at (650‒500 cm^‒1^) in the low-frequency region 31. In addition, the X-ray photoelectron spectroscopy (XPS) results clearly showed the existence of the Fe‒O bond in the FerH (**Figure S10**). Specifically, the presence of Fe^2+^ ion indicates the formation of Fe‒O coordination bonds owing to the reduction of Fe centers with the electrons donated from the catechol π-systems. In the O 1s XPS spectra, the decreased binding energy of O‒C from 533.5 eV to 531.8 eV contributes to O‒C interacting with Fe^3+^ ions [38‒40] The ζ potential of FerH maintained at ~ -25 mV, the negative ζ potential is beneficial for the dispersion of nanocomplex in biological-related conditions due to reducing the cellular uptakes and the immune system opsonization 27. In addition, the negative ζ potential benefits the efficient extracellular cluster formation with the free Fe^3+^ addition, which directly reduces the rate of metabolism and induces prolonged retention time in the tumor (**Figure S11**).

## Photothermal conversion of FerH

The ultraviolet-visible-near-infrared (UV-Vis-NIR) absorption spectrum of FerH displayed a broad absorption ranging from 400 to 1000 nm, which was attributed to the ligand-to-metal charge transfer (LMCT) in the HMT-Fe^3+^ complexes (**Figure 2C**). The wavelengths at the absorption peak of FerH stayed the same with varied ratios of Fe^3+^ to HMT from 1:1 to 1:5 (**Figure S12**). The strong NIR adsorption of FerH allows an efficient penetration of photons for therapeutic photothermal conversion. The absorption intensity increased over time indicating the continuous formation of FerH nanoaggregates, which agrees with the size-growing tendency (**Figure 2D**). Theoretical calculations were carried out to study the HOMO-LUMO gaps of various FerH complexes with optimized geometries (**Figure S13**). The different coordinates (**S2**, **S4**, **S7**) possessed HOMO-LUMO gaps of 2.7, 1.8, and 0.5 eV, respectively, which are lower than that of HMT (5.76 eV), which supported the presence of a broad absorption band in the visible-to-NIR region. (**Figure 2E and Figure S14**).

When the tissue temperature increases to 42‒52 °C, effective PTT causes rapid cell death due to microvascular thrombosis and ischemia occurring 2. Our FerH nanoaggregates afforded the solution temperature to 63 °C with a concentration of 300 μg mL^‒1^ under irradiation (808 nm, 1 W cm^‒2^), which demonstrated the promise as an effective PTA (**Figure 2F and Figure S15**). To achieve the optimal therapeutic effects and avoid the damages from the high temperature, 150 μg mL^‒1^ was chosen as the concentration for further evaluation. In addition, the FerH solution exhibited a high photostability with negligible attenuation of peak solution temperatures after five “On-Off” cycles of laser irradiation (**Figure 2G**). The photothermal conversion efficiency of FerH nanoaggregates was calculated as 60.2% (**Figure S16**), which was the record-high the previously-reported MPN photothermal nanosystems including Fe-(-)-epigallocatechin-3-gallate-gossypol (FeEG) 28, Fe-tannic acid (FeTA) 29, Vanadium-TA (VTA) 29, and Ruthenium-TA (RuTA) 29, Fe-anthocyanins (FeAP) 30, Fe-gallic acid (FeGA) 31, and other PTAs like, Fe_3_O_4_ 41, Au nanorods (AuNR) 42, black phosphorus (BP) 43, Au-ZIF8 44, graphene oxide (GO) 45, although not as high as graphene 45 (**Figure 2H**).

## *In vitro* performance of FerH

**Figure 3:**
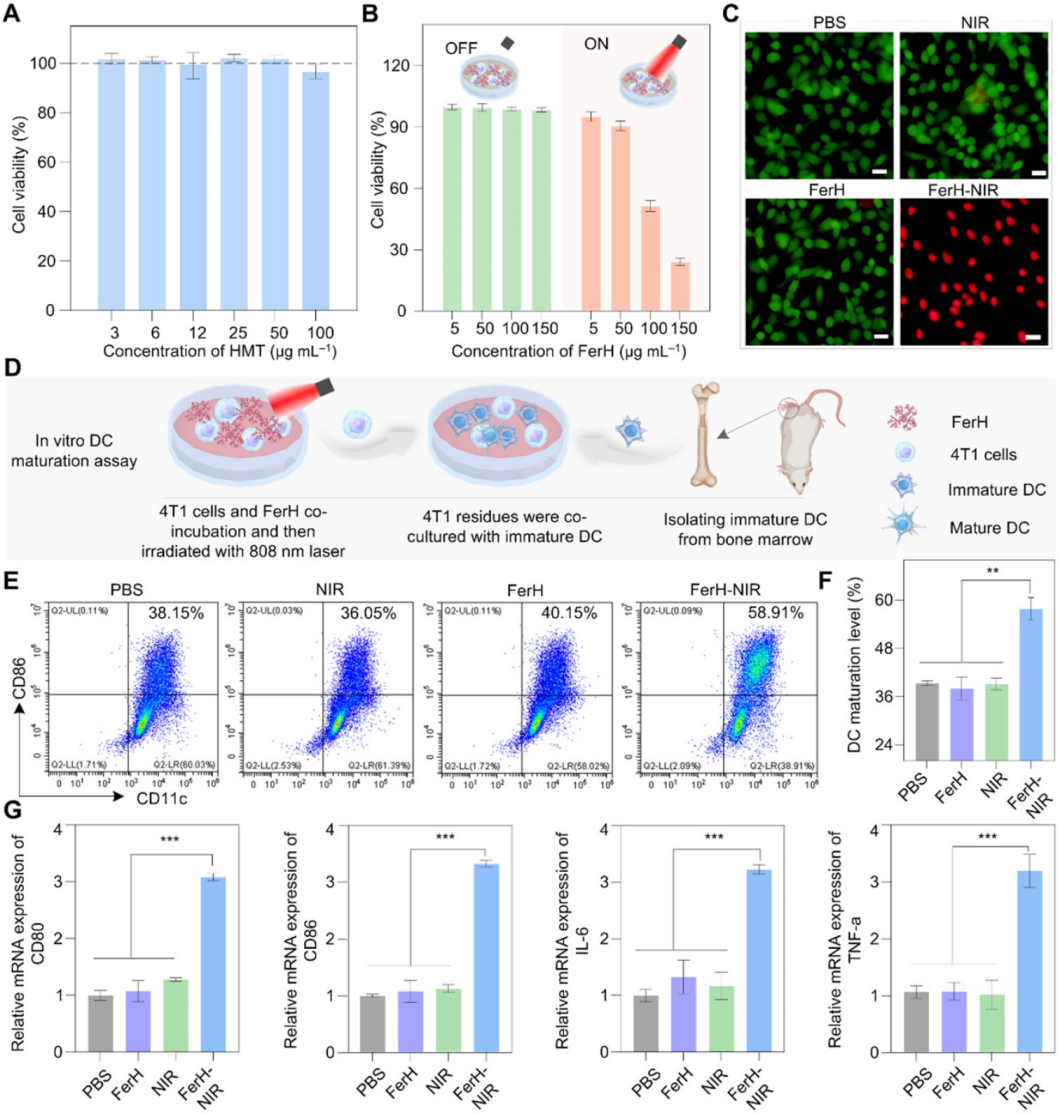
***In vitro* photothermal therapy and DCs maturation induced by FerH-mediated photothermal and enhanced immunotherapeutic therapy. (A)** The cell viability of 4T1 cells was detected by CCK8 assay after incubation with HMT (3‒100 µg mL^-1^) for 24 h. Data are presented as mean ± SD. (n = 3). *(B)* Cytotoxicity of FerH on 4T1 cells with or without laser irradiation (808 nm) for 10 min. Data are presented as mean ± SD. (n = 3). **(C)** Fluorescence images of calcein- Acetoxymethyl Ester (green, live cells) and PI (red, dead cells) co-stained 4T1 cells treated with PBS, 808 nm laser irradiation alone, FerH alone, and the combination of FerH and laser, respectively. Scale bars, 5 µm. **(D)** Schematic illustration of *in vitro* DCs maturation evaluation. **(E)** Flow cytometry analysis of CD11c and CD86 in BMDCs after different treatments. **(F)** Quantitative analysis of DCs maturation level. **(G)** Relative mRNA expression levels in DCs, including CD80, CD86, TNF-α, and IL-6. For (F-G), data are presented as mean ± SD. (n = 3). Statistical significance was calculated via one-way ANOVA with Tukey's multiple comparisons (*p < 0.05, **p < 0.01, ***p < 0. 001).

The cytotoxicity of FerH nanoaggregates was evaluated by CCK8 assay and flow cytometry with 4T1 cell lines. The results revealed that free HMT (**Figure 3A**) was nontoxic to tumor cells at a wide concentration range. The groups treated with PBS, FerH, and NIR also showed a neglected effect, while the cells were necrotic or apoptotic when treated with FerH under laser irradiation (808 nm) (**Figure 3B-C, Figure S17, and Figure S18**). Considering that iron in FerH may induce ferroptosis, we then investigated the reactive oxygen species (ROS) level on FerH-treated cells, the little difference in the ROS level revealing that FerH didn't induce ferroptosis (**Figure S19**). Therefore, FerH possesses high biocompatibility without inherent toxicity and shows photocytotoxicity upon laser irradiation.

Importantly, we also noticed that PTT can induce immunogenic cancer cell death, when ablating by hyperthermia, dying cancer cells release damage associated molecular patterns (DAMPs) and tumor-associated antigens. PTT-generated DAMPs include surface-expressed calreticulin (CRT), extracellular adenosine triphosphate (ATP), high mobility group B1 (HMGB1), and heat shock protein 90 (HSP90). Once exposed to heat damage, which acts as immunostimulatory factors to activate the host immune system against cancer by stimulating antigen presentation of dendritic cells (DCs) 4, 46. As the most powerful APCs, dendritic cells (DCs) capture and cross-present the antigens released by tumor cells and then activate T cells, thus initiating antitumor immune responses. Then DCs undergoes maturation with the secretion of proinflammatory cytokines, including tumor necrosis factor-α (TNF-α) and interleukin-6 (IL-6) 47, 48. The expression of co-stimulatory molecules (CD80 and CD86) are the hallmarks of DCs maturation. The percentage of DCs maturation (CD11c^+^, CD86^+^) and cytokines secretion were analyzed by flow cytometry and real-time quantitative polymerase chain reaction (RT-qPCR) (**Figure 3D**). The post-irradiation FerH triggered the maturation of DC by 57%, while FerH alone or laser irradiation merely prompted the DC maturation level (**Figure 3E-F**). Meanwhile, when compared with the FerH group, the secretion of IL-6 and TNF-α cytokines dramatically increased by 2.9-fold and 2.5-fold after combined with laser irradiation. These results indicated that FerH-mediated PTT significantly induced immunogenic cancer cell death, and then DMAPs activated DCs and enhanced DC cross-presenting through DC maturation, suggesting the FerH-mediated PTT augmented immunotherapeutic effect (**Figure 3G**).

## *In vivo* studies of FerH self-assembly

**Figure 4:**
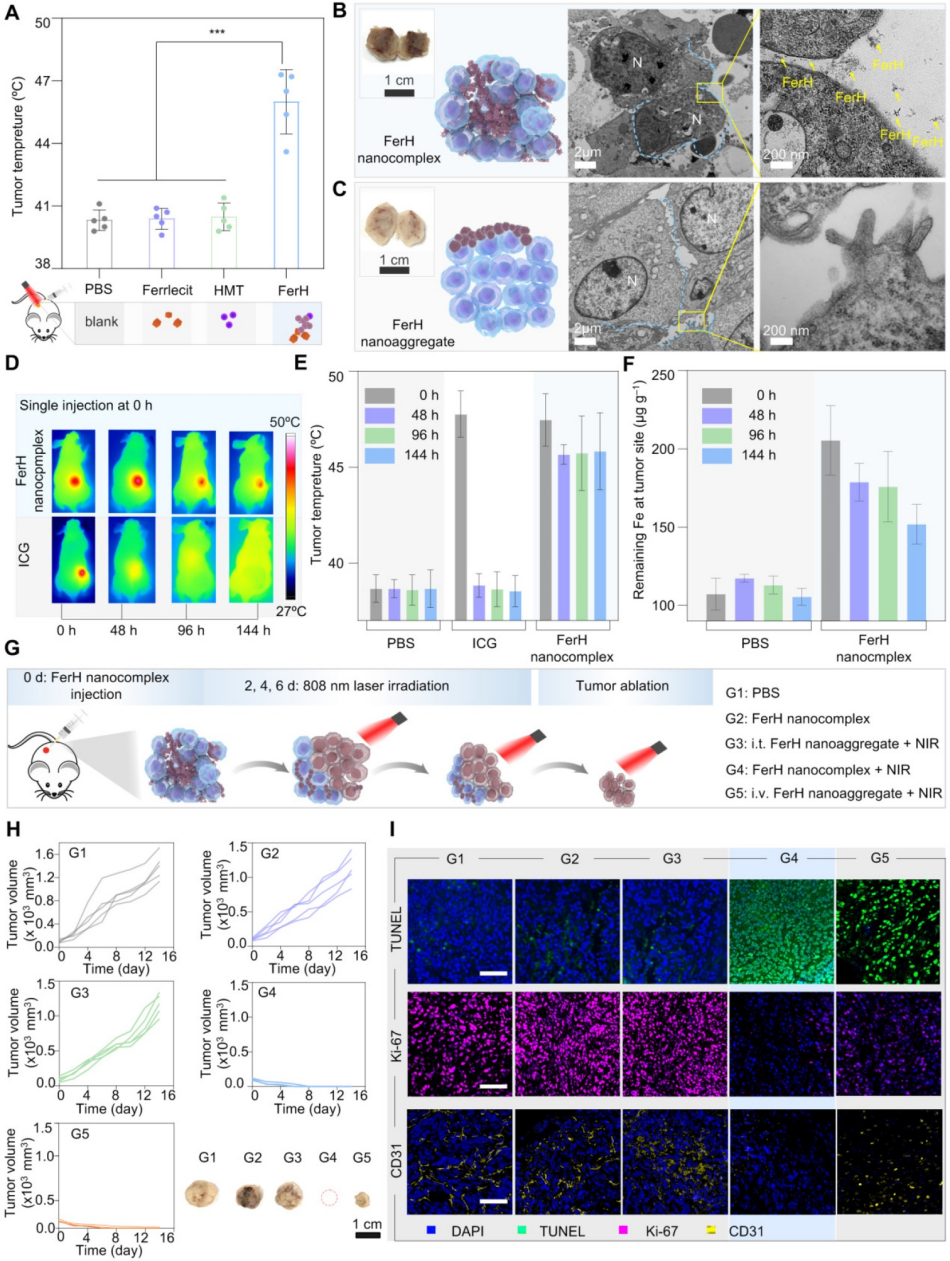
***In vivo* evaluation on FerH nanocomplexes assembly and photothermal therapy inhibited tumor growth. (A)** Comparison of mean temperature in tumor region under 808 nm laser irradiation. Mice received orthotopic administration of Ferrlecit™, HMT, and FerH nanocomplex, respectively, before laser irradiation. Data are presented as mean ± SD. (n = 5). Statistical significance was calculated via one-way ANOVA with Tukey's multiple comparisons. **(B)** TEM images of FerH nanocomplexes and **(C)** FerH nanoaggregates treated tumor tissues, tumors were excised 24 h post orthotopic administration FerH. N, nucleus. Blue line, cell membrane. The yellow arrow indicates the formation of FerH nanoaggregates. Scale bars, 2 µm (left), 200 nm (right). Inset, photographs of excised tumors. Scale bars, 1 cm. **(D)** Representative IR thermal images of nude mice bearing subcutaneous 4T1 tumors at different time points. The mice were orthotopic injection of ICG and FerH nanocomplexes, respectively, then irradiated with an 808 nm laser (1 W cm^-2^) for 10 min. All images were obtained from nude mice that received ICG (30 µg mL^-1^) and FerH nanocpmplex (150 µg mL^-1^). Different batches of mice were used for each time point tracking. **(E)** The corresponding average temperature in the tumor region under laser irradiation at 0, 48, 96, and 144 h post single injection. Data are presented as mean ± SD. (n = 3). **(F)** Measurement of Fe^3+^ ions levels of tumors. The tumors were excised at 0, 48, 96, and 144 h post single injection. The retention ability of FerH was measured using a spectrophotometric assay at the wavelength of 520 nm. Data are presented as mean ± SD. (n = 8). **(G)** Schematic of intratumoral self-assembled FerH mediated photothermal therapeutic approach in experimental groups with different treatment settings. FerH nano-complexes or aggregates were injected every 2 days for G1, G2, and G3. The 808 nm laser irradiation was applied for 10 min for each PTT. For G4, FerH nanocomplexes were injected once on the first day, followed by laser irradiation only on the following therapeutic days. The body weight and tumor volume were recorded every 3 days during the therapeutic period. i.v.: intravenous injection, i.t.: intratumoral injection. **(H)** 4T1 tumor growth curves of each mouse after different treatments and representative tumor images of each group. Scale bar, 1 cm. **(I)** TUNEL, Ki-67, and CD31 staining tumors after various treatments. Scale bars, 50 µm.

An FDA-approved iron supplement (Ferrlecit™) was selected as the source of Fe^3+^ ions in the following *in vivo* studies. FerH nanocomplexes were subcutaneously injected into the 4T1 tumors on nude mice immediately after the mixing of HMT and Ferrlecit™ solutions. Upon irradiation, the temperature of the tumor region reached 50 °C in the group treated with FerH nanocomplexes, while the other groups had negligible temperature rise (**Figure 4A and Figure S20**), indicating the successful coordination of Fe^3+^ ions and HMT in the tumor.

To further ascertain the penetration of FerH nanocomplexes within tumor tissues, we performed an *ex vivo* analysis. It can be distinguished that the tumor treated with FerH nanocomplexes turned dark entirely (**Figure 4B, inset**)**,** while only a partially darkened region was observed within the tumors injected with aged FerH nanoaggregates (**Figure 4C, inset**). The results confirmed that the small-sized FerH nanocomplexes penetrated through the whole tumor in contrast to the limited diffusion of the large-sized FerH nanoaggregates after aging. The tumor treated with FerH nanocomplexes showed abundant aggregation of FerH nanoaggregates in the extracellular matrix, while no such observation was obtained in the tumor treated with large-sized FerH nanoaggregates (**Figure 4B-C**), indicating that the discrete FerH nanocomplexes is favorable of the diffusion into tumor microstructures. Furthermore, energy dispersive X-ray spectroscopy (EDS) mapping and Fe element quantitative analysis (**Figure S21**) indicated that the relative content of Fe in the tumors treated with FerH nanocomplexes was 30% higher than in the PBS group. These results suggest the deep diffusion and the subsequent retention of the FerH nanoaggregates in tumors.

Prolonged retention of FerH nanoaggregates was compared with a clinically available NIR contrast agent, indocyanine green (ICG). The mean temperature of the tumors treated with ICG decreased throughout the measuring time (48 °C at 0 h, 37 °C at 48 h, 37 °C at 96 h, and 37 °C at 144 h) (**Figure 4D-E**), indicating low levels of ICG retention within tumor over time. In contrast, the tumor group applied with a single administration of FerH nanocomplex seeds showed no significant temperature difference (48 °C) even after 144 h (6 days). Further evidence for this retention capability was provided by the measurement of the Fe^3+^ ion content in tumors. In the FerH nanocomplex seeds group, the remaining Fe^3+^ ion concentration in tumor tissue was 180 μg mL^‒1^ even the 144 h after injection, which was much higher than that of the control groups (110 μg g^‒1^) (**Figure 4F**). The major organs at different times post-FerH injection were collected for* ex vivo* Fe content analysis, and the result indicated that FerH could be metabolized by the liver and kidney (**Figure S22**).

## FerH-mediated PTT to inhibit tumor growth

*In vivo* therapeutic effects of FerH-mediated PTT were subsequently evaluated in the animal groups (G1‒G5) with different treatments (**Figure 4G**). The results indicated that the FerH alone without NIR irradiation could not inhibit the growth of 4T1 tumors (G2, **Figure 4H, and Figure S23**). The intravenous injection of aged FerH nanoaggregates (G3) cannot inhibit tumor growth owing to the non-target accumulation at tumor sites. By applying FerH nanocomplexes intratumorally under NIR irradiation, mice in G4 stayed tumor-free and no further relapsing in the subsequent 14 days (**Figure S24**). For the mice treated with FerH nanoaggregates and the same parameter NIR irradiation (G5), it was observed that tumors were not completely ablated (**Figure 4H**). These results suggest that the limited penetration of aged FerH nanoaggregates may leave tumor residues after the PTT. Hence, the efficient PTT on tumors was achieved by combining the deep penetration of FerH nanocomplex seeds and the prolonged retention of assembled nanoaggregates.

The subsequent analysis of the body weight displayed no significant difference among groups, which indicated no obvious side effects caused by the FerH treatments (**Figure S25**). The H&E stained images of major organs (heart, liver, spleen, lung, and kidney) showed neglectable differences (**Figure S26**). The biomarkers, including albumin (ALB), alanine transaminase (ALT), creatinine (CREA), and urea (UREA) (**Figure S27**), also showed no significant difference among different groups, which demonstrates the high biocompatibility of FerH-mediated PTT.

To unveil the apoptosis and proliferation levels of the tumor cells, excised tumors were stained with terminal deoxynucleotidyl transferase-mediated dUTP nick end labeling (TUNEL) and Ki-67. The highest positive TUNEL signals were observed with G4 samples (**Figure 4I**), revealing that FerH-based PTT effectively induced tumor cell death. The strongest Ki-67 signals of G4 suggested the inhibition of tumor proliferation by FerH-based PTT. In addition, tumor blood vessels were stained with the anti-CD31 antibodies. The minimal yellow area of G4 indicated that FerH-based PTT inhibited the proliferation of tumor blood vessels. These results suggest that FerH nanocomplexes could be synthesized *in vivo* and performed a dynamic size-changeable property within the microstructures of solid tumors, which leads a desired therapeutic PPT effects.

## Inhibition of distant tumors by PTT-induced immunotherapy

**Figure 5:**
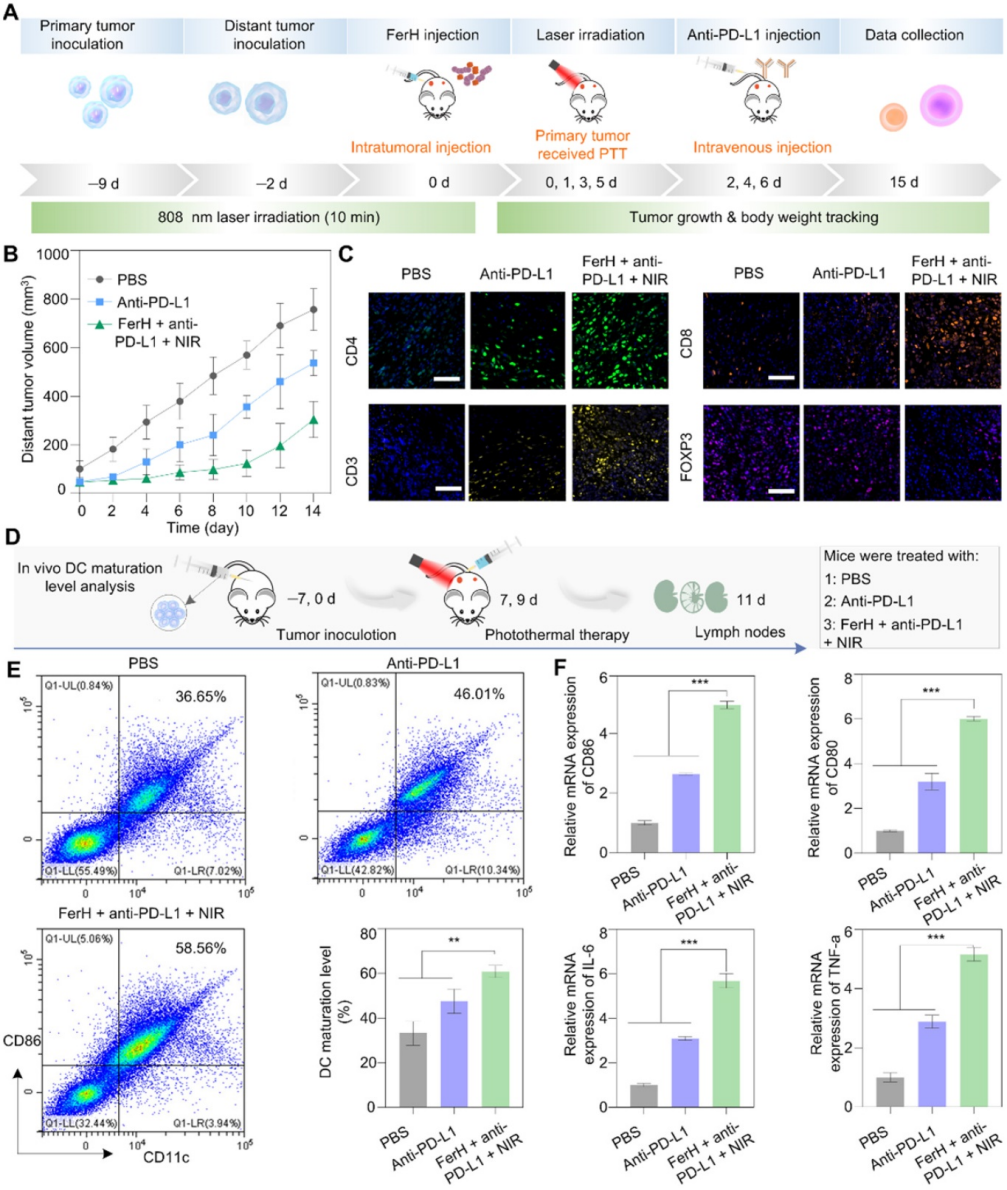
***In vivo* photothermal immunotherapy. (A)** Schematic illustration of the distant tumor treatment schedules. **(B)** The volume of distant tumors from the 4T1 tumor-bearing mice. Data are presented as mean ± SD. (n = 5). **(C)** Immunofluorescence investigation of distant tumor tissues after treatment with the following markers: CD3, CD4, CD8, FOXP3 (green: CD4, yellow: CD3, orange: CD8, pink: Treg, blue: nuclei). Scale bar, 50 µm.** (D)** Schematic illustration of the *in vivo* DC maturation evaluation. **(E)** Flow cytometry analysis of CD11c and CD80 (gated on CD11c^+^) in tumor-draining lymph nodes after different treatments and quantitative analysis of *in vivo* DC maturation level, experiments performed in triplicate with similar results. **(F)** Relative mRNA expression in DCs and serum, including CD80, CD86, TNF-α, and IL-6. For (E-F), data are presented as mean ± SD. (n = 3). Statistical significance was calculated via one-way ANOVA with Tukey's multiple comparisons (*p < 0.05, **p < 0.01, ***p < 0. 001).

Immunotherapy induced by PTT was verified when co-administrated with an immune checkpoint blockade antibody to eradicate metastatic cancer cells. The pathway block of the interactions between the PD-1 and PD-L1 by the antibodies (*i.e.* anti-PD-L1) has emerged as a powerful strategy in immune activation 48, 49. PD-1 is a transmembrane protein that is highly expressed on tumor-specific T cells. PD-L1 is expressed by tumor cells and works to escape anti-tumor responses when conjugating with PD-1, thereby suppressing the functions of T cells. Monoclonal antibodies (e.g., anti-PD-L1) that block this pathway have emerged as powerful weapons for cytotoxic T cells to function effectively. To investigate the potential of photothermal immunological synergism *in vivo*, we combined FerH-mediated PTT with an anti-PD-L1 antibody to inhibit the distant tumors with a dual-tumor mouse model (**Figure 5A**). Compared with the rapid growth of tumors in the PBS group, the anti-PD-L1 group exhibited a moderate inhibition effect, due to the insufficient infiltration of TME. Noteworthy, the group treated with FerH nanocomplexes coupled with anti-PD-L1 under NIR irradiation significantly suppressed the tumor proliferation with an inhibition rate of 59.8% (**Figure 5B**), which indicated that FerH-mediated PTT combined with anti-PD-L1 could achieve a synergistic effect for the inhibition of distant tumors beyond laser irradiation.

To reveal the in-depth mechanisms of the antimetastatic efficacy resulting from the combinational use of FerH nanocomplexes and anti-PD-L1, the intratumoral infiltration of T lymphocytes (CD3), including cytotoxic T lymphocytes CTLs (CD8) and T helper cells (CD4) was investigated by the immunofluorescence staining. Strong fluorescence signals for CD3, CD4, and CD8 cells were found in the groups treated with FerH nanocomplexes and anti-PD-L1 under NIR irradiation (**Figure 5C**). Regulatory T cells (T_reg_, immunosuppressive T lymphocytes) are recruited by tumor cells to inactivate CTLs that hamper the antitumor immune response 47. Forkhead box P3-positive (FOXP3) as the marker of Tregs, we detected Tregs infiltration. The group treated with FerH nanocomplexes and anti-PD-L1 under NIR irradiation exhibits the strongest fluorescence intensity, suggesting that combined with αnti-PD-L1, FerH-based PTT effectively strengthened T-cell infiltration of tumors.

Furthermore, by measuring the DC maturation levels in tumor-draining lymph nodes (**Figure 5D**), we evaluated the immunological response initiated by FerH-mediated PTT. DC maturation was promoted by 58.5% with the FerH mediated PTT combined with anti-PD-L1 (**Figure 5E, Figure S28**), which was 1.6-fold higher than that of the anti-PD-L1 group. The relative expression of CD80 and CD86 genes were also 1.8-fold and 1.9-fold higher than that of the group treated with anti-PD-L1 alone (**Figure 5F**). Meanwhile, the highest immune cytokines (IL-6, TNF-a) were also observed in the group treated with the combined agents. Taken together, our results suggest that FerH-based PTT triggered immunogenicity could be enhanced by combining with anti-PD-L1, thus contributing to the inhibition of distant tumors.

## Conclusions

In summary, we reported a size-changeable metal-phenolic supramolecular nanoaggregate (FerH) simply based on the coordination of a natural polyphenol HMT and Fe^3+^ ions released from a commercial iron supplement. The discrete FerH nanocomplexes can penetrate the microstructures of solid tumors and form interconnected nanoaggregates through an *in vivo* pseudo-stepwise kinetic self-assembly process. Specifically, the two aromatic rings in the HMT molecules have different electron densities for Fe^3+^ ion coordination. The coordination preference of Ring A leads to the rapid consumption of existing Fe^3+^ ions to form nanocomplexes with a lag period, while further coordination with Ring B assists the growth of nanocomplexes into aggregates. The prolonged retention of 6 days was achieved in animal experiments due to the formed nanoaggregates subsequently, which, coupled with a record-high photothermal conversion efficiency (60.2%), realized the ablation of the primary tumors upon a single-dose administration. FerH-mediated PTT accelerated immune activation and substantially augmented the therapeutic effects for the distant tumors when co-administrated with anti-PD-L1. This work provides a new strategy based on dynamic metal-phenolic supramolecular chemistry in the development of transformable nanomedicines to improve the PTT efficacy against solid tumors.

## Experimental Methods

### Preparation of FerH complex

All solutions were freshly prepared for immediate use. Specifically, 10 μL HMT (20 mg mL^‒1^) was first added to 1.8 mL of water and vortexed for 10 s, then, 10 μL FeCl_3_·6H_2_0 (8.9 mg mL^‒1^) was added and the dispersion was vortexed for 10 s, and the color of the suspension turned dark blue immediately after the addition of metal solution. Next, the pH of the solution was adjusted by adding 200 μL PBS (100 mM, 7.4) and vortexed for 10 s. Finally, the dispersion was directly used for photothermal testing. In all experiments, the concentration of FerH was defined by the total weight of HMT and FeCl_3_·6H_2_O in FerH suspension.

### Synthesis of sodium ferric gluconate complex

Sodium ferric gluconate complex was synthesized as follows. Firstly, the 2.5 g D-sodium gluconate was dissolved in 10 mL ultrapure water, and then 10 mL 1.18 M Na_2_CO_3_ was added. Then, a solution containing FeCl_3_·6H_2_O (1.25 mol L^‒1^, 12 mL) was added dropwise under continuous stirring. The pH of the solution was adjusted to 12 by 5 M NaOH solution. The mixture was heated at 100 ℃ for 3 h in an oil bath, filtered, and then cooled to room temperature. After that, the complex was precipitated by 0.05 L ethanol, and then centrifuged at 4000 r min ^‒1^ for 10 min, the precipitate was collected and dried in a vacuum to obtain the sodium ferric gluconate complex crude. The crude was redissolved in 10 mL distilled water and then precipitated in ethanol, the supernatant was decanted and the precipitate was washed with 10 mL ethanol, followed by 10 mL acetone to obtain refined sodium ferric gluconate complex.

### The size of FerH tracking

To investigate the *in vitro* growing behavior of FerH. 10 μL HMT (10 mg mL^‒1^) was dispersed in 1mL PBS (pH 7.4). Then, 10 μL FeCl_3_·6H_2_O (8.9 mg mL^‒1^) was added and the size of nanocomplex was monitored meanwhile by dynamic light scattering (Malvern Zetasizer Nano ZSP) lasted for about 100 min without interruption.

### Synthesis of Fe-Gallic acid nanodots (FeGA)

The FeGA was synthesized according to the reported methods 31. Briefly, 66 mg of PVP was dissolved in 8.8 mL of water at room temperature under vigorous stirring. A FeCl_3_ aqueous solution (0.2 mL, 100 mg mL^‒1^) was then added to the aqueous PVP solution. After 1 h of incubation, a gallic acid aqueous solution (1 mL, 10 mg mL^‒1^) was added to the above reaction mixture and stirred overnight. The resulting nanodots were dialyzed (MWCO (molecular weight cut off) ¼ 25000) against deionized water for 24 h. The photothermal conversion efficiency of FeGA was calculated to be 58.3%.

### Data fitting

To model the growth kinetics, we attempt to fit the data with exponential functions. A single exponential enables us to fit the data well, producing a reduced r-squared value of greater than 0.95. The ln(D) versus *t* exhibited a linear relationship suggesting a first-order of the size increase kinetics. The r-squared value is found as 0.93. Fitting of the DLS data was performed using Origin (OriginLab, Northampton, MA) software.

### Computational details

All complexes have been optimized by Perdew-Burke-Ernzerhof hybrid functional (PBE0) method and def2-SVP basis set with Grimme's DFT-D3(BJ) empirical dispersion correction by Gaussian 09 package 50. Harmonic vibrational frequency was performed at the same level to confirm that imaginary frequency is absent in the molecules, *i.e.*, they locate at the minima of the potential energy surface. Moreover, water has been introduced as implicit solvents by the SMD (Solvation model density) solvation model, and the HOMO (Highest Occupied Molecular Orbital) and LUMO (Lowest Unoccupied Molecular Orbital) of these complexes were obtained by combining Multiwfn 3.7 51 and VMD 1.9.3 52 software, whose input files were extracted by Gaussian checkpoint file.

### Investigation of photothermal performance

To confirm the optimal concentration, 1 mL FerH suspensions with varying concentrations were irradiated using with 808nm laser (1 W cm^‒2^). The blank group (pure DI water) was also investigated to make a comparison. The laser-caused temperature increase was monitored by a Fluke Ti400 thermal imaging camera. For the photothermal stability test, 1 mL FerH suspension (150 μg mL^‒1^) was irradiated with an 808 nm laser (1 W cm^‒2^) for five heating/cooling cycles. The photothermal conversion efficiency of FerH was measured according to the previous report 53. Typically, FerH suspension was placed in a 1.7 mL centrifuge tube, then the suspension was irradiated with an 808 nm laser (1 W cm^‒2^) until the temperature rose to a peak followed by the removal of the laser and a subsequent natural cooling process. The temperature during laser irradiation and cooling was monitored by a Fluke Ti400 thermal imaging camera. Photothermal conversion efficiency (*η*) is calculated according to Eq (1) as follows:


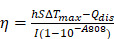

(1)

Where h means the heat transfer coefficient, S refers to the surface area of the container, ΔT_max_ is the maximum temperature change, and Q_Dis_ is the heat-related to the light absorbance of the DI water, which is 0 mW. I stand for laser power density. A808 represents the absorbance value of the sample at 808 nm. hS is determined based on Eq (2) as follows:


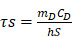

(2)

Where m_D_ is the mass (1 g) and C_D_ is the heat capacity (4.2 J g ^‒1^) of pure water. To determine τ_s_, the following Eq (3) is required:


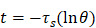

(3)

Where θ is the ratio of _Δ_T to _Δ_T_max_. From the inset of Figure S16, τ_s_ is calculated to be 168 s.

### Electrospray ionization mass spectrometry (ESI-MS) of FerH

For the ESI-MS test, FerH was prepared at two methods, one was prepared and stood for 7 days in aqueous condition, and the other was prepared under vigorous stirring for 7 days. Then collected the FerH complex for ESI-MS analysis.

### Cells lines

CHOK1 cells were maintained in DMEM (Invitrogen, USA) containing 10% fetal calf serum (Invitrogen) and 1% penicillin/streptomycin at 37 °C in a 5% CO_2_ atmosphere. 4T1 cells were placed in Roswell Park Memorial Institute 1640 (RPMI 1640, Invitrogen) containing 10% fetal calf serum (Invitrogen) and 1% penicillin/streptomycin at 37 °C in a 5% CO_2_ atmosphere.

### *In vitro* cell viability assay

For cytotoxicity measurement, 4T1 cells and CHOK1 cells were seeded in a 96-well plate (at the density of 10^4^ cells per well) and incubated overnight. Then the medium was refreshed and the cells were co-incubated with various drugs (HMT, FerH) with different concentrations for 24 h, followed either with or without NIR laser irradiation at a power density of 1 W cm^‒2^ for 10 min. The washing process with PBS was performed in all experiments after incubation laser irradiation. Finally, cell viability was detected using the CCK8 assay. All experiments were independently performed three times.

### Live/dead cell staining

4T1 cells were seeded in a 96-well plate incubated overnight and cocultured with or without FerH for 24 h. Then the cells were further sequentially irradiated with an 808 nm laser (1 W cm^‒2^, 10 min) at room temperature. After that, the cells were co-incubated with both calcein-AM (2 × 10^‒6^ M) and PI (4 × 10^‒6^ M) for 30 min in a humidified atmosphere containing 5% CO_2_ at 37 °C. Finally, the cells were washed three times with PBS and observed by an optical microscope.

### Intracellular ROS detection

4T1 cells were seeded in 6-well plates (10^5^ cells per well) and incubated overnight. Then, the cells were treated with HMT, FerH nanocomplex (150 μg mL^-1^), and FerH nanoaggregate (150 μg mL^-1^) for different periods. DCFDA (10 × 10^-6^ M) was added to plates for 30 min staining. After washing with cool PBS, the fluorescence of cells was obtained using a Thermo Scientific Varioskan Flash multimode reader.

### Flow cytometry experiments

4T1 cells were seeded in a 6-well plate and incubated overnight, then co-cultured with FerH (150 μg mL^‒1^) for 24 h. Subsequently, the cells were irradiated with an 808 nm laser (1 W cm^‒2^, 10 min). After that, the cells were washed with PBS. Then the cells were stained with Annexin V-FITC/PI Apoptosis Detection Kit according to the manufacture instruction and tested by CytoFLEX LX (Beckman).

### *In vitro* promotes the maturation of dendritic cells (DCs)

Mouse immature DCs were prepared as previously described protocol 32. Briefly, Sterile surgical scissors were used to extract the tibias, femurs, and humeri of BALB/C mice. Isolated bones were submerged in 75% ethanol, rinsed with PBS, and then transferred to a separate PBS solution. In a sterile environment, both ends of the bones were carefully trimmed to expose the bone marrow. Bone marrow cells were isolated by flushing the bones with sterile PBS and centrifuged (650 g) for 6 min. Followed by filtering the bone marrow through a 70 μm nylon web and gathering the filtrate. Next, red blood cells were lysed by incubation with ACK lysis buffer for 5 min at room temperature. And then, the cells were washed and centrifuged 2 times, resuspended in 1640 medium (Invitrogen) containing IL-4 (10 ng mL^‒1^) and GM-CSF (20 ng mL^‒1^). On day 3, the culture was replaced with the fresh medium (containing the same concentration of GM-CSF, and IL-4 as before) and the non-adherent cells were removed. On day 6, the non-adherent cells were harvested for further use. To investigate FerH-based PTT-induced maturation of DCs, 4T1 cells were incubated overnight, then 4T1 cells were treated with PBS, FerH nanocomplex with or without laser irradiation. Subsequently, residual 4T1 cells after treatments were co-incubated with harvested immature DCs in a 6-well plate. After being co-cultured for 24 h, DCs were stained with FITC-CD11c monoclonal antibody, PE-CD86 monoclonal antibody, and APC-CD80 monoclonal antibody following the instruction. Finally, DCs were analyzed by flow cytometry.

### *In vivo* DCs maturation assay

The 4T1 tumor-bearing mice were randomly divided into four groups (as described in the article). When the tumor volume reached ≈100 mm^3^ on day 7, the mice received treatment on day 7 and day 9. Then on day 11, lymph nodes of each mouse were individually collected and triturated into a single-cell suspension. After that, the cells were stained with FITC-conjugated anti-mouse CD11c, APC anti-mouse CD 80, and PE-conjugated anti-mouse CD86 at 4 °C for 30 min and dispersed in 500 μL of PBS after centrifugation. Then the cells were analyzed by flow cytometry.

### RT-qPCR assay

BMDCs/tumor-draining lymph nodes were collected. Total RNAs were extracted by Triquick Reagent (Trizol Substitute, Solarbio, China). RNA (500 ng), quantified by NanoDrop2000 (Thermo Fisher Scientific, USA), was reversely transcribed to cDNA using the first-strand cDNA synthesis kit (Vazyme, China). Quantitative PCR was applied using the SYBR Green dye (Vazyme, China) on quant studio 3 applied biosystems (Thermo Fisher Scientific, USA). All primers were synthesized by Tsingke Biotechnology and their sequences were listed in Supplementary Table S1. The parameters of PCR assays were shown as follows: initial denaturation at 95 °C for 30 s, 40 cycles of denaturation at 95 °C for 10 s, and primer annealing and reaction at 60 °C for 30 s. Comparative quantification was assessed using the 2^-ΔΔCt^ method with glyceraldehyde-3-phosphate dehydrogenase (GAPDH) as the endogenous control.

### Self-assembly tests of FerH *in vivo*

For the assembly test of FerH in tumor tissue, the 4T1 tumor-bearing 6 weeks-old nude mice were randomly divided into four groups (5 mice per group). After the mice received different treatments for 1 h, the mice were anesthetized and the tumor regions were irradiated with laser (808 nm, 1W cm^‒2^) for 10 min, the temperature was recorded by a Fluke Ti400 thermal imaging camera. For the FerH retention test, randomly divided three groups of nude mice were treated with PBS, ICG, and FerH nanocomplex respectively. After ICG and premixed FerH injection on the first day, the temperature of each mouse was monitored every 2 days by a Fluke Ti400 thermal imaging camera.

### TEM tumor samples preparation

The tumor tissues were prefixed with a 3% glutaraldehyde, then the tissue was postfixed in 1% osmium tetroxide, dehydrated in series acetone, infiltrated in Epox 812 for a longer, and embedded. The semithin sections were stained with methylene blue and ultrathin sections were cut with a diamond knife and stained with uranyl acetate and lead citrate. Finally, tumor sections were examined with JEM-1400-FLASH Transmission Electron Microscope.

### Measurement of iron content in tumor and major organs

The tumors and major organs (heart, liver, spleen, lung, kidney) of mice were collected and weighed. Then digested in 3 M hydrochloric acid/10% trichloroacetic acid at 65 °C for 20 h. After that, 100 μL of each acid extract was mixed with 1 mL glycine/hydrochloric acid buffer (pH 2.8), and 100 μL ferrozine disodium salt chromogen reagent. After 20 minutes of incubation at room temperature, the absorbance at 520 nm was measured using a Thermo Scientific Varioskan Flash multimode reader.

### *In vivo* photothermal therapy

Female 4T1 tumor-bearing Balb/C mice were randomly divided into five groups when the tumor volume reached 100 mm^3^, then the mice received different treatments. The tumor size was measured with a vernier caliper, and the weight of the mice was measured every other day during two weeks treatment period. The tumor volume was calculated following the equation: V=L × W^2^/2, where L is the length of the tumor, and W is the width of the tumor. The serum of each mouse was collected for biochemical analysis. The major organs were harvested and fixed in 4% paraformaldehyde for H&E staining. Noteworthy, on day 5, extra 3 tumors of each group were harvested for immunofluorescence staining (Ki-67, CD31, TUNEL). For the PTT-induced immunotherapy, 4T1 cells were implanted subcutaneously on the left and right flanks of BALB/C mice for an interval of one week. Subsequently, the left tumor received PTT.

### *In vivo* photothermal immunotherapy

Balb/c female mice were given one week to acclimatize to the facility environment, followed by the establishment of the tumor model. 5 × 10^6^ 4T1 cells were injected under the skin near the thighs on the left side. After seven days, the average tumor size reached ~ 80 mm^3^, followed by the injection of 2.5 × 10^6^ 4T1 cells on the right side. After 2 days of the second tumor inoculation, mice were divided into three groups: (1) PBS; (2) anti-PD-L1; (3) FerH + NIR + anti-PD-L1. Tumors on the left side were regarded as primary tumors for PTT treatment, and the tumors on the right side were the secondary tumors without PTT treatment. For FerH + NIR + anti-PD-L1 group, FerH nanocomplex (150 μg mL^-1^) was injected intratumorally on the first therapeutic day (day 0) and continuous NIR (808 nm, 1W cm^-2^) irradiation was then applied for 10 min. On days 2, 4, and 6, only NIR irradiation was conducted for FerH + NIR + anti-PD-L1 group. The anti-PD-L1 antibody (750 mg kg^-1^) was subsequently injected intravenously on days 1, 3, and 5 in the anti-PD-L1 group and FerH + NIR + anti-PD-L1 group. Tumor volumes were measured every 2 days.

### Statistical analysis

All the results are reported as mean ± SD. The differences among groups were analyzed using one-way ANOVA analysis with Tukey's multiple comparisons; (*) P < 0.05, (**) P < 0.01, ***p < 0.001). All statistical differences were calculated by using GraphPad Prism 8.0 (GraphPad Software, Inc., CA, USA). In all types of statistical analysis values of P < 0.05 were considered significant.

## Supplementary Material

Supplementary materials and methods, figures and table.
