# Supplementary material for: Intratumoral synthesis of transformable metal-phenolic nanoaggregates with enhanced tumor penetration and retention for photothermal immunotherapy
Source: Theranostics. 2022 Aug 29;12(14):6258–72. doi: 10.7150/thno.74808 (PMC9475467; doi:10.7150/thno.74808)
Supplement: Supplementary file 1 — Supplementary materials and methods, figures and table. [file thnov12p6258s1.pdf]

Supplementary Information

Intratumoral synthesis of transformable metal-phenolic nanoaggregates with enhanced tumor penetration and retention for photothermal immunotherapy

Xianglian He[1], Hongfu Zhu[2], Jiaojiao Shang[1], Meifeng Li[1,3], Yaoyao Zhang[1,4], Shicheng Zhou[1,5], Guidong Gong[1,*], Yunxiang He[1,*], Anna Blocki[6], Junling Guo[1,7,8,*]

[1]BMI Center for Biomass Materials and Nanointerfaces, College of Biomass Science and Engineering, Sichuan University, Chengdu, Sichuan 610065, China.

[2]Collage of Material Science and Engineering, Sichuan University, Chengdu, Sichuan 610065, China.

[3]Department of Biomass Chemistry and Engineering, College of Biomass Science and Engineering, Sichuan University, Chengdu, Sichuan 610065, China.

[4]Key Laboratory of Birth Defects and Related of Women and Children of Ministry of Education, Department of Pediatrics, The Reproductive Medical Center, Department of Obstetrics and Gynecology, West China Second University Hospital, Sichuan University, Chengdu, Sichuan 610041, China.

[5]Department of Bioengineering, School of Engineering, The University of Tokyo, 7-3-1 Hongo, Bunkyo-ku, Tokyo 113-8656, Japan.

[6]Institute for Tissue Engineering and Regenerative Medicine, The Chinese University of Hong Kong (CUHK), Shatin, Hong Kong SAR, China.

[7]State Key Laboratory of Polymer Materials Engineering, Sichuan University, Chengdu, Sichuan 610065, China.

[8]Bioproducts Institute, Department of Chemical and Biological Engineering, University of British Columbia, Vancouver, BC V6T 1Z4, Canada.

*E-mail: guidong@scu.edu.cn (G.G.); yxhe@scu.edu.cn (Y.H.); junling.guo@scu.edu.cn, junling.guo@ubc.ca (J.G.)

Contents

Materials and animals 3
Characterization 3
Tables...5
Supplementary figures...6
References..34

Materials and animals

All chemicals and reagents were used as obtained without further purification unless otherwise mentioned. Iron (III) chloride hexahydrate ($FeCl_3·6H_2O$), hematoxylin, sodium carbonate, sodium hydroxide, ethanol, glycine, and acetone were purchased from Titan (China). D-sodium gluconate and 3-(2-Pyridyl)-5,6-bis(4-sulfophenyl)-1,2,4-triazine disodium salt hydrate (ferrozine disodium salt) was purchased from Aladdin (China). Phosphate buffered saline (PBS) and normal saline were purchased from Gibco (USA). Recombinant murine interleukin-4 (IL-4) and animal-free recombinant murine granulocyte-macrophage colony-stimulating factor (GM-CSF) were purchased from Peprotech (USA). FITC-CD11c monoclonal antibody (clone N418, Catalog: 11-0114-82), PE-CD86 monoclonal antibody (clone GL1, Catalog: 12-0862-81), and APC-CD80 monoclonal antibody (clone 16-10A1, Catalog: 11-0801-81) were purchased from Thermo Fisher Scientific (USA). Anti-PD-L1 antibodies were purchased from Bioxcell (USA). Cell Counting Kit-8 (CCK8) and Calcein/PI Live/Dead assay kit were purchased from Beyotime (China). Annexin V-FITC/PI Apoptosis Detection Kit was purchased from Dalian Meilun Biotech Co., Ltd (China). High-purity Milli-Q (MQ) water with a resistivity of 18.2 MΩ cm was obtained from an inline Millipore RiOs/Origin water purification system.

Female 8-week-old Balb/c mice and nude mice were purchased from Dashuo Laboratory Animal Technology, Ltd. (China). Mice were kept in the Sichuan University West China Medical Center animal facility. Before any experiment, mice had at least one week to acclimatize to the facility environment. All the experiments and procedures were performed in accordance with the National Institutes of Health guide for the care and use of Laboratory Animals (NIH Publications No. 8023, revised 1978). All animal experiments were approved by the Experimental Animal Management and Ethics Committee of West China Second University Hospital (Approval number: KS2021632).

Characterization

The size distribution and zeta potential of the FerH nanocomplexes and nanoaggregates were obtained by dynamic light scattering (Malvern Zetasizer Nano ZSP). The nanocomplex morphology and structure were acquired with a transmission electron microscope (Tecnai G2 F20 S-TWIN). Fourier transform infrared (FTIR) spectroscopy was performed from 4000 to 400 nm^{-1} with Thermo scientific Nicolet iS10, USA. Raman spectrum was obtained with Raman Spectrometer (Thermo Fisher DXR 2xi) at the wavenumber range of 100–3700 nm^{-1} and excitation at 1064 nm. The ultraviolet near-infrared absorption spectra were measured by UV-vis-NIR spectrometry (PE1050, Perkin-Elmer). The photothermal temperature of suspension and tumor was recorded by Fluke thermal camera (Ti400). Flow cytometry was measured on CytoFLEX LX (Beckman). Scanning electron microscope (SEM) images of tumor tissue and energy dispersive spectroscopy (EDS) mapping were conducted on a scanning electron microscope (ZEISS Gemini 300). Elemental

analysis of the FerH was conducted by energy-dispersive X-ray spectroscopy (aAXIS Ultra DLD). Transmission electron microscopy (TEM) images of tumors were obtained by JEM-1400-FLASH (JEOL) transmission electron microscope. TEM images of FerH at different time points were acquired by Tecnai G2 F20 S-TWIN with an operation voltage of 200 kV. The electrospray ionization mass spectrometry (ESI-MS) was carried out on an Agilent 6550 QTOF mass spectrometer with a positive ion source of 4000 V voltage and gas temperature of 300 °C. The drying gas was set as 150 °C with a flow rate of 15 L min^{-1}. The sheath gas temperature was set as 350 °C with a flow rate of 12 L min^{-1}. The mass range was 50 to 3000 m z^{-1}. Cyclic voltammograms were acquired by employing an electrochemical workstation (CHI600E, Shanghai Chenghua). Fluoresce images of 4T1 cells with Calcein and PI staining were obtained by CLSM (Leica STELLARIS).

Table S1. Primers for RT-qPCR

**Primers**	**Primer sequence**	
	Forward (5′-3′)	Reverse (5′-3′)
**CD80**	ACCCCCAACATAACTGAGTCT	TTCCAACCAAGAGAAGCGAGG
**CD86**	CTGGACTCTACGACTTCACAATG	AGTTGGCGATCACTGACAGTT
**IL-6**	GTTCTCTGGGAAATCGTGGA	TGTACTCCAGGTAGCTA
**TNF-α**	AGACCCTCACACTCAGATCA	TCTTTGAGATCCATGCCGTTG

Supplementary figures

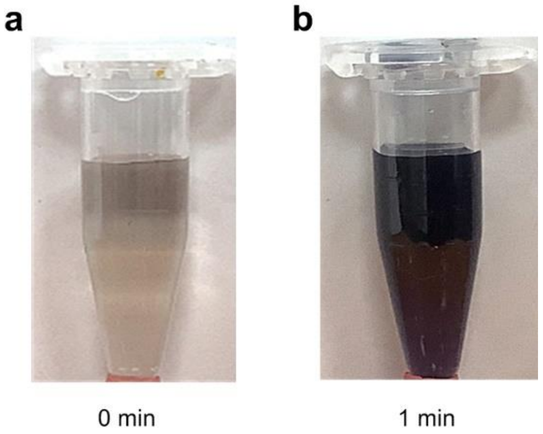

Figure S1. The appearance of FerH solution with aging. (**a**) Light brown solution of nanocomplexes by the coordination of HMT and Fe^{3+} ions. (**b**) Dark blue nanoaggregates formed by the coordination-induced self-assembly.

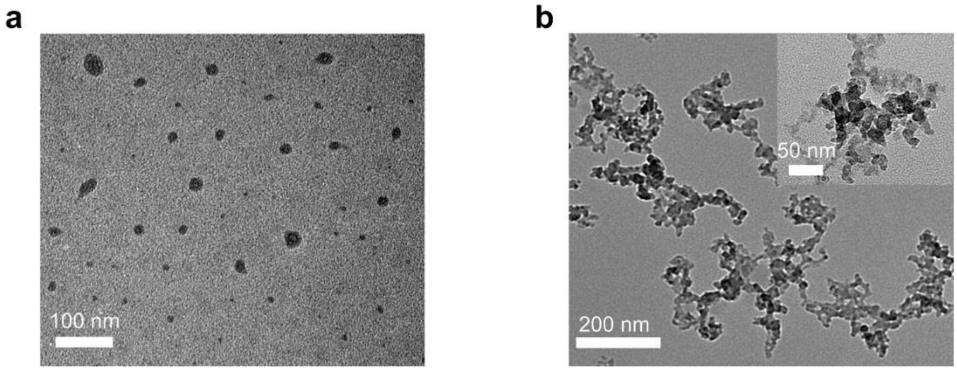

Figure S2. TEM images of FerH at different time points. (**a**) The nanocomplex seeds of FerH after mixing HMT and Fe^{3+} ions within 30 min. Scale bar, 100 nm. (**b**) TEM images of FerH cluster after mixing HMT and Fe^{3+} ions for 3 h. Scale bar, 200 nm, and 50 nm (inset).

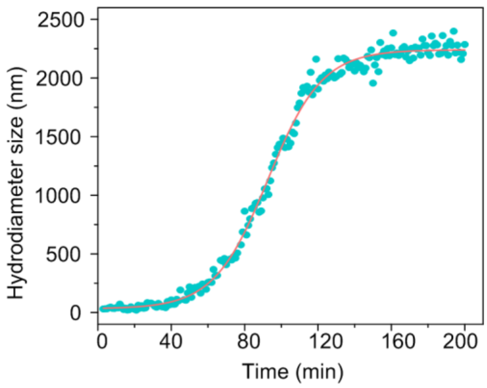

Figure S3. The hydrodiameter size of FerH during the self-assembly, the growth of FerH tends to reach a plateau at 200 min.

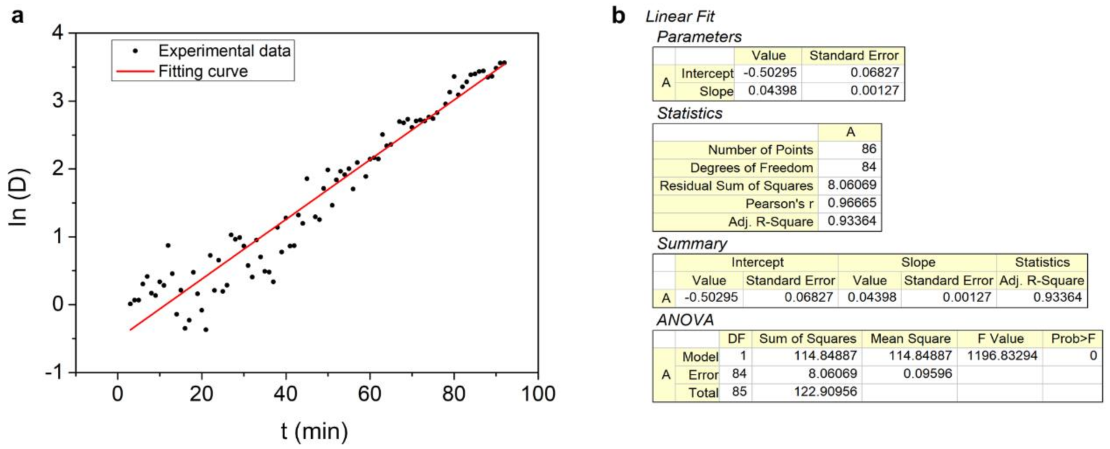

Parameters

		Value	Standard Error
A	Intercept	-0.50295	0.06827
	Slope	0.04398	0.00127

Statistics

	A
Number of Points	86
Degrees of Freedom	84
Residual Sum of Squares	8.06069
Pearson's r	0.96665
Adj. R-Square	0.93364

Summary

	Intercept		Slope		Statistics
	Value	Standard Error	Value	Standard Error	Adj. R-Square
A	-0.50295	0.06827	0.04398	0.00127	0.93364

ANOVA

		DF	Sum of Squares	Mean Square	F Value	Prob>F
A	Model	1	114.84887	114.84887	1196.83294	0
	Error	84	8.06069	0.09596		
	Total	85	122.90956			

Figure S4. DLS data processing for kinetic studies. (**a**) Linear fitting of ln(D) vs. *t* for the FerH growth kinetics. D: hydrodiameter size of FerH. (**b**) Fitting results by Origin software.

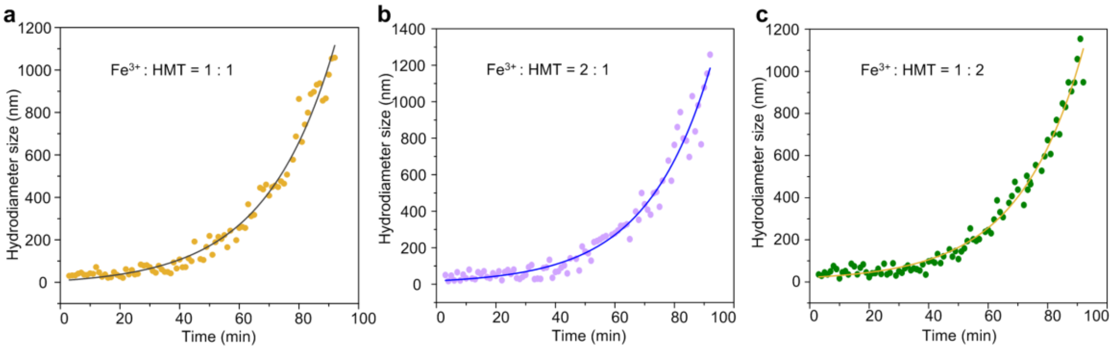

Figure S5. Hydrodiameter size of FerH prepared by the varied molar ratio of Fe^{3+} ions to HMT at (a) 1:1, (b) 2:1, and (c) 1:2 during the self-assembly.

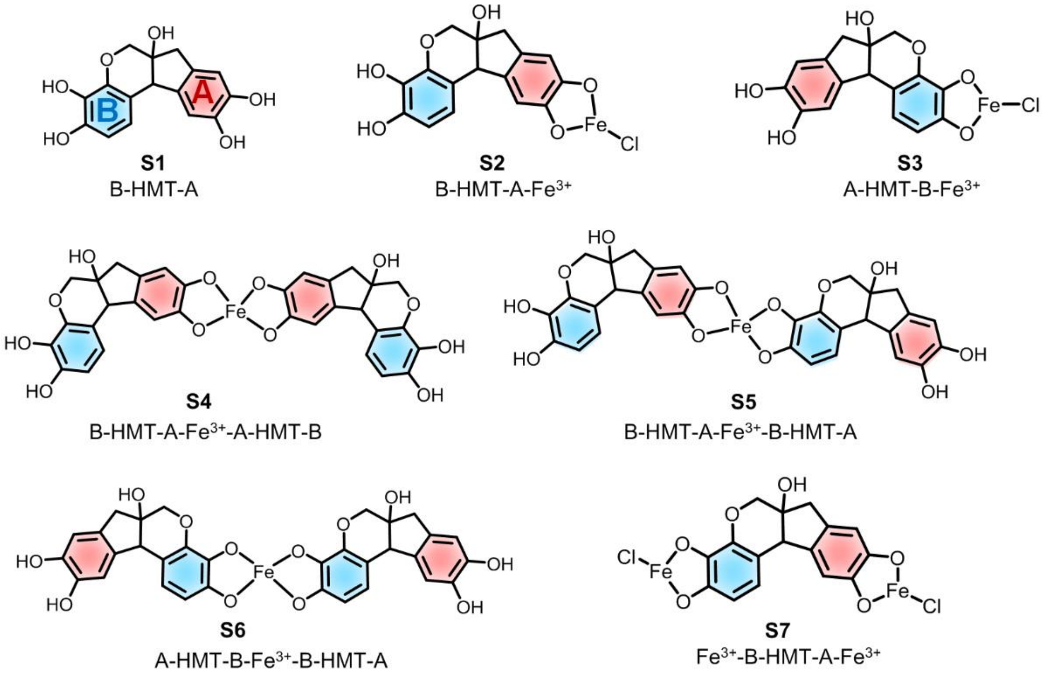

Figure S6. Proposed coordinated complexes of HMT and Fe^{3+} ions at a molar ratio of 1:1. Ring A and Ring B of HMT have different reactivities with Fe^{3+} ions to provide different structures of coordinated subunits.

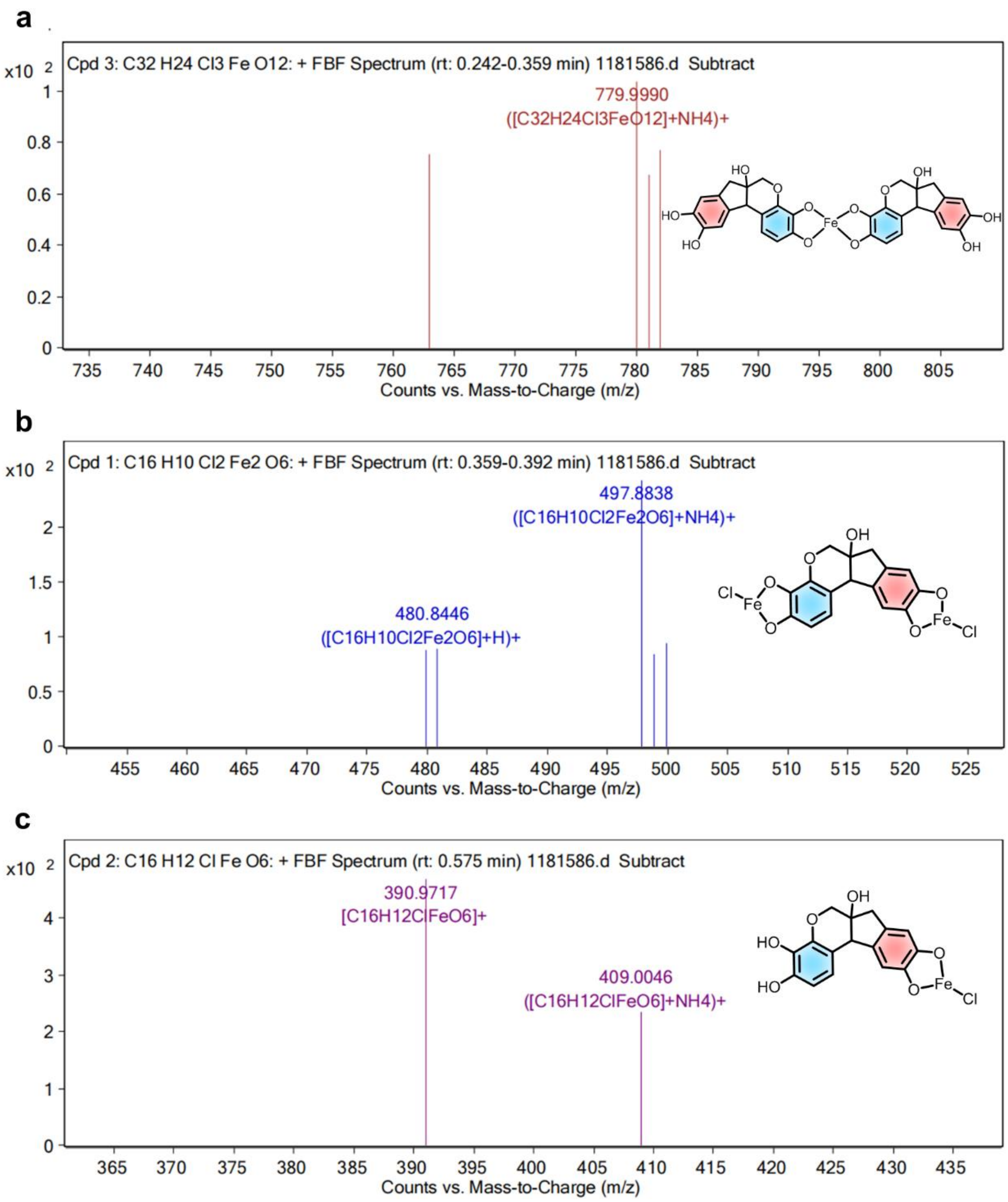

Figure S7. ESI-MS spectra of obtained FerH coordination complexes. HMT coordinates with Fe^{3+} ions at molar ratios of (**a**) 2:1, (**b**) 1:2, and (**c**) 1:1.

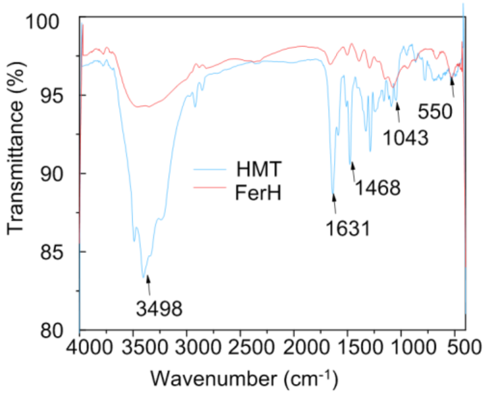

Figure S8. Coordination characterization by FTIR spectroscopy. The FTIR spectra of (**a**) HMT (red) and (**b**) FerH nanocomplexes (blue). The strong FTIR band of FerH at 550 cm^{-1} is characteristic of the Fe-O vibrations [1], indicating the successful formation of Fe-O coordination in FerH.

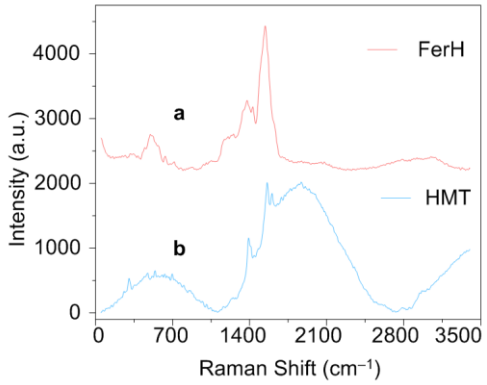

Figure S9. Coordination characterization by Raman spectroscopy. Raman spectra of (**a**) FerH (red) and (**b**) HMT (blue).

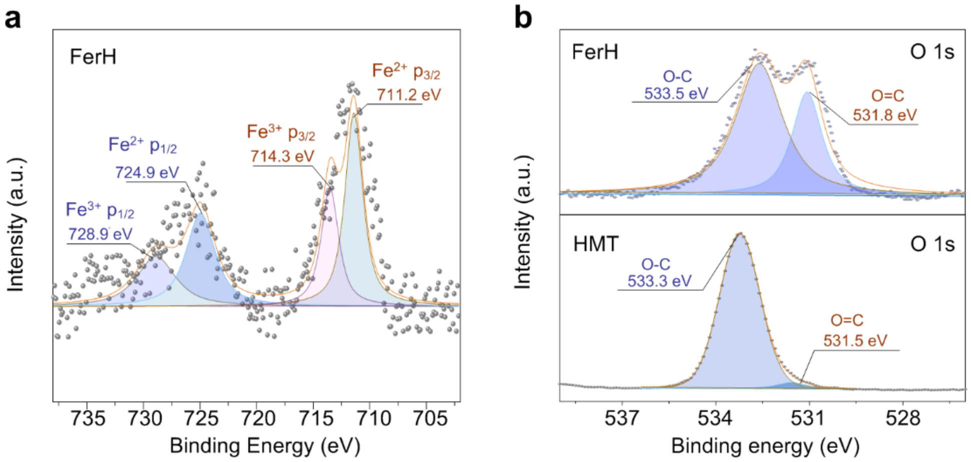

Figure S10. Coordination characterization by XPS. (**a**) Fe 2p spectra of FerH (**b**) O 1s spectra of FerH and HMT.

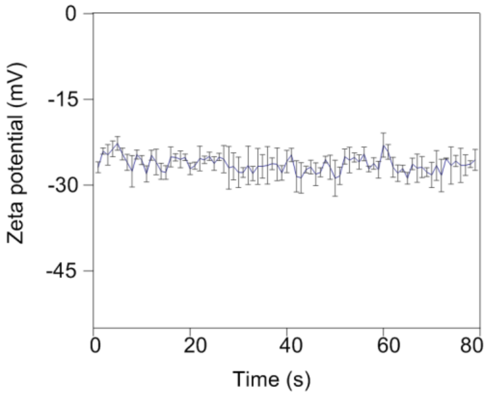

Figure S11. Time-dependent zeta potentials of FerH nanocpmplex.

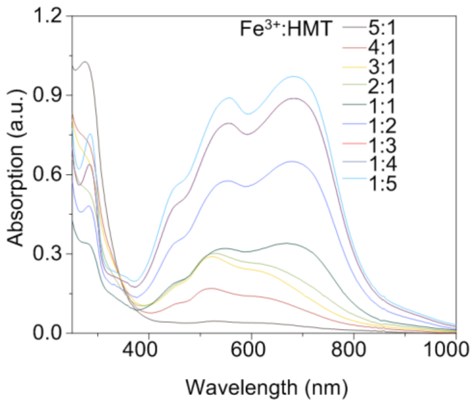

Figure S12. The absorption spectrum of FerH prepared by Fe^{3+} to HMT at different ratios. The maximum absorption wavelengths stayed the same with varied ratios from 1:1 to 1:5.

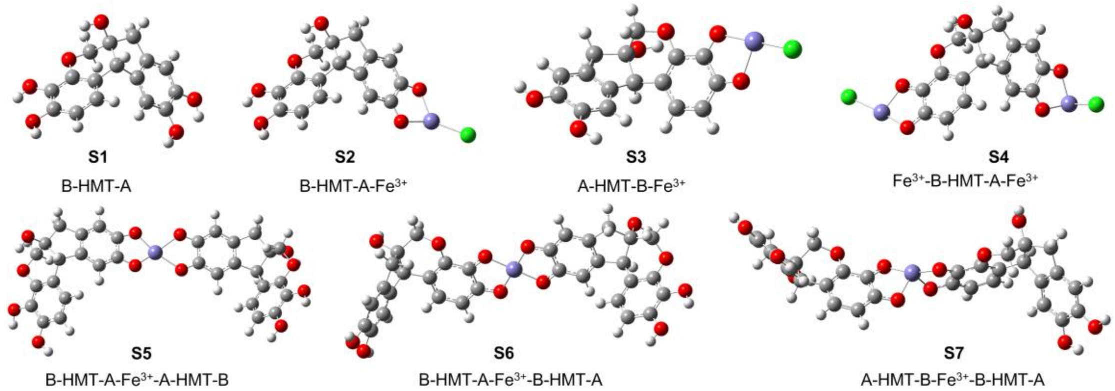

Figure S13. Calculated structures of different coordination compounds. Optimized geometries of HMT (**S1**), B-HMT-A-Fe^{3+} (**S2**), A-HMT-B-Fe^{3+} (**S3**), B-HMT-A-Fe^{3+}-A-HMT-B (**S4**), B-HMT-A-Fe^{3+}-B-HMT-A (**S5**), A-HMT-B-Fe^{3+}-B-HMT-A (**S6)**, and Fe^{3+}-B-HMT-A- Fe^{3+} (**S7**). The white, gray, red, green, and purple colors denote H, C, O, Cl, and Fe atoms.

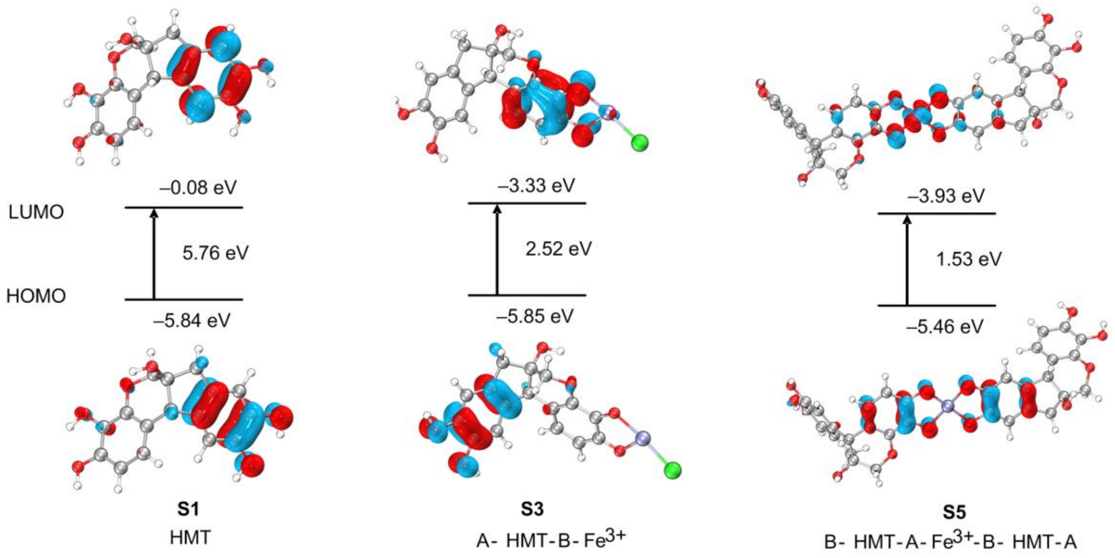

Figure S14. Computational studies on the HOMO-LUMO gaps of different coordination compounds. HMT (**S1**) possesses a large gap of 5.76 eV. After coordination with Fe^{3+} ions, the complexes all exhibited decreased HOMO-LUMO gap energy with 2.53 eV and 1.84 eV for A-HMT-B-Fe^{3+} (**S3**) and B-HMT-A-Fe^{3+}-B-HMT-A (**S5**), respectively. The white, gray, red, green, and purple colors denote H, C, O, Cl, and Fe atoms. The red and cyan colors denote positive and negative phases of orbitals whose isovalues are equal to 0.05.

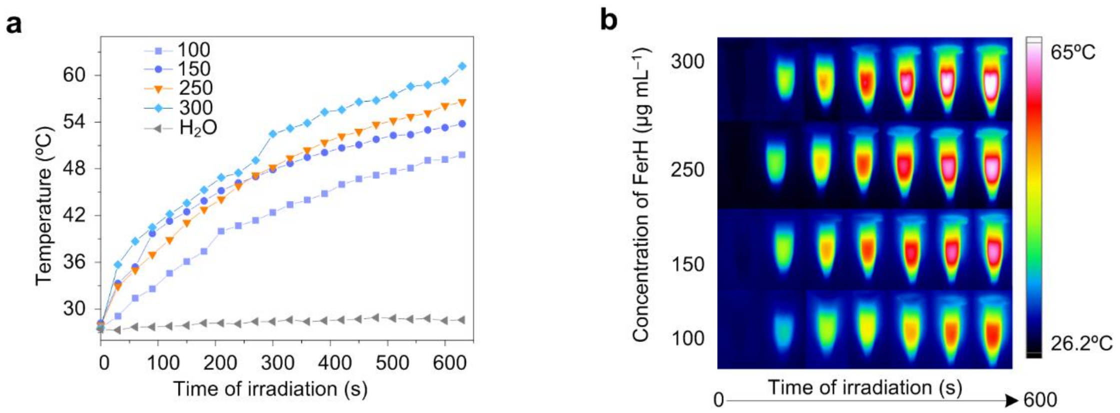

Figure S15. Characterization of photothermal conversion property. (**a**) Temperature increasing curve of FerH at different concentrations (100–300 μg mL^{-1}) under laser irradiation (808 nm, 1 W cm^{-2}) for 10 min. (**b**) Thermal images of FerH at different concentrations under laser irradiation for 10 min.

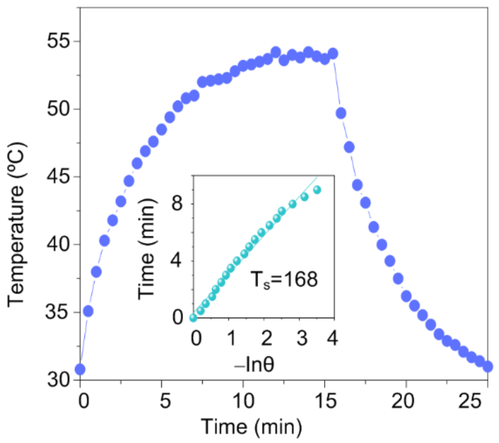

Figure S16. The cooling curve of FerH (150 µg mL^{-1}). The inset shows time constant (τ_S) for the heat transfer from the system were determined by applying the linear time data from the cooling period.

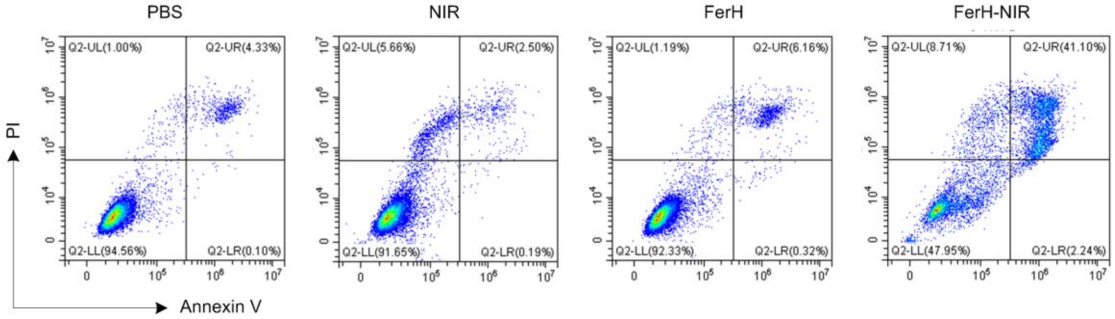

Figure S17. Flow cytometric apoptosis analysis of Annexin V-FITC/PI stained 4T1 cells after treated with FerH complexes at different conditions. FerH performed cytotoxicity only with the co-presence of FerH and NIR irradiation.

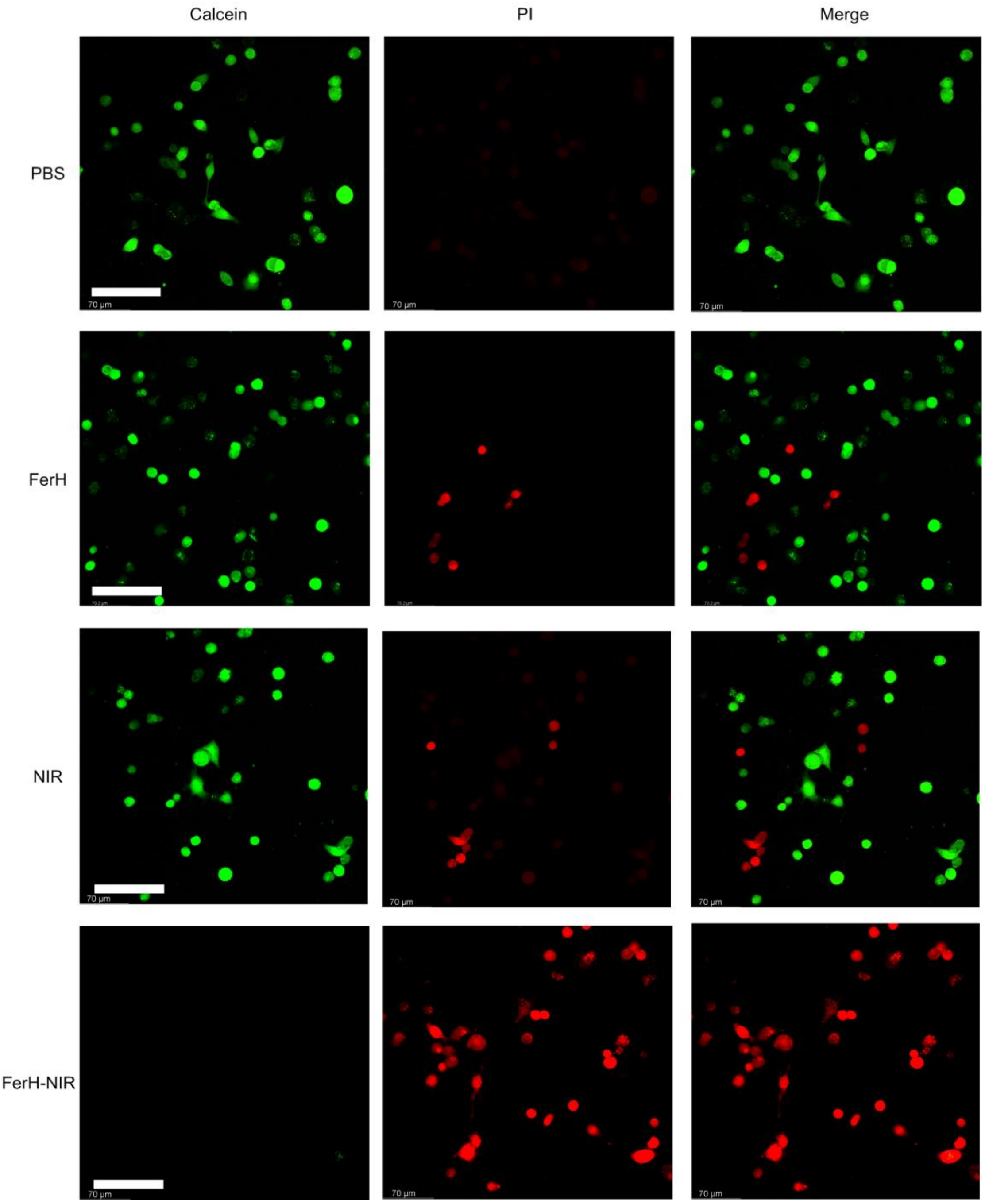

Figure S18. Fluorescence images of calcein (green, live cells) and PI (red, dead cells) co-stained 4T1 cells treated with PBS, FerH alone, 808 nm laser irradiation alone, and the combination of FerH and laser, respectively. Scale bars, 70 µm

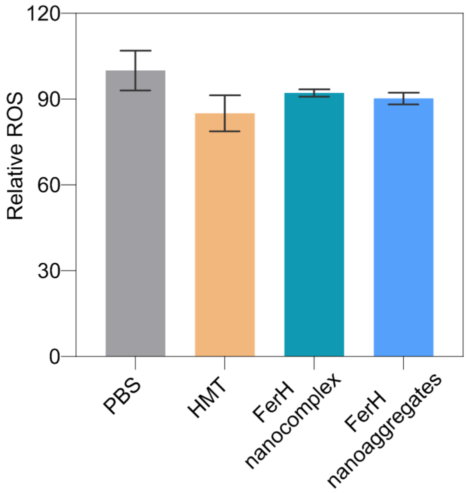

Figure S19. Quantification of total ROS by using 2,7-dichlorodi-hydrofluorescein diacetate fluorescent probes; n = 3.

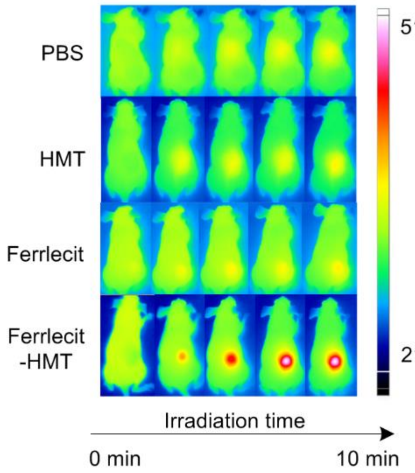

Figure S20. Characterization on photothermal effects in vivo. Thermal images of tumor region under laser irradiation (808 nm, 1W cm^{-2}) for 10 min after treatments in mice.

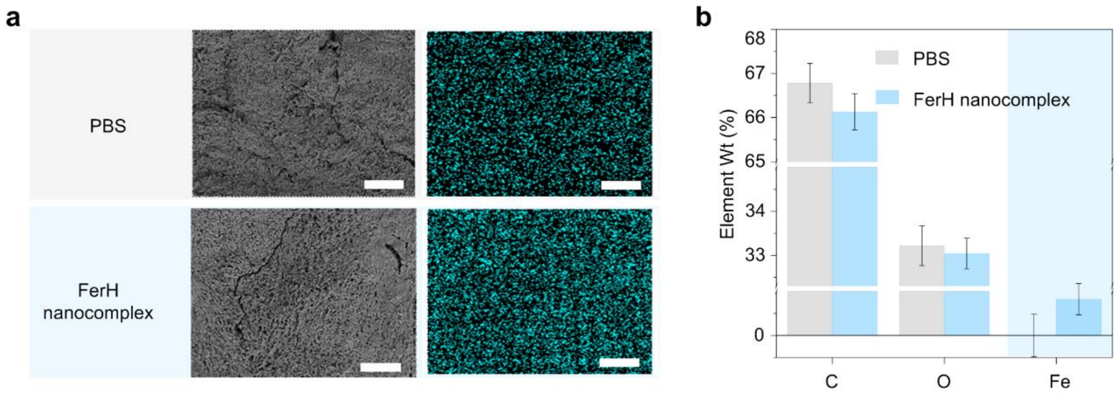

Figure S21. SEM characterization on the Fe^{3+} ion distribution in tumor tissues. (**a**) SEM images and Fe element mapping of tumor section with different treatments. scale bar 100 µm. (**b**) Corresponding EDS analysis of C, O, Fe elements. Wt (%), weight percentage. Data are presented as mean ± SD. (n = 3).

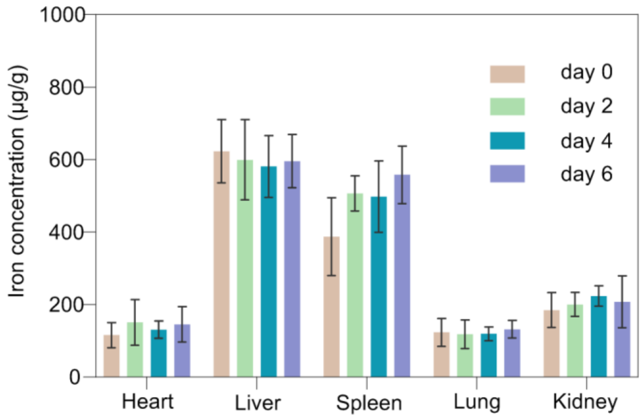

Figure S22. ***Ex vivo*** **biodistribution of iron in tumor and major organs of mice bearing 4T1 tumor at day 0, 2, 4, and 6 post-FerH nanocomplex injection. Each point corresponds to mean ± s.d., n = 4.**

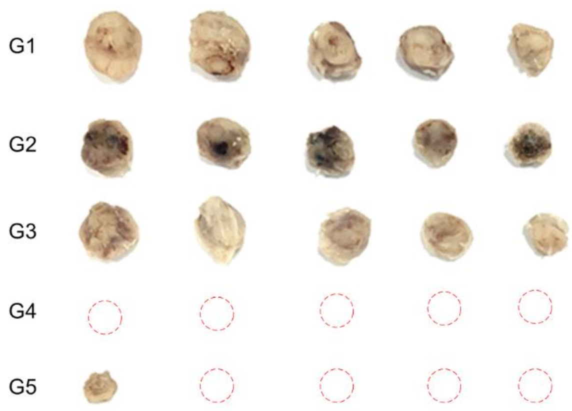

Figure S23. Photothermal therapy effects with FerH on tumor growth. Photographs of tumors harvested after different treatments in 14 days.

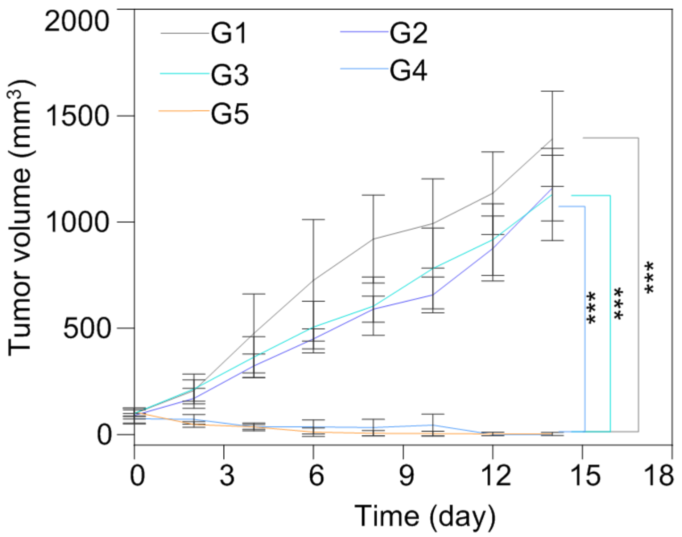

Figure S24. Relative tumor volume after different treatments on day 14. G1: intratumoral injection PBS, G2: intratumoral injection FerH nanocomplex, G3: intravenous injection FerH cluster + NIR, G4: intratumoral injection FerH nanocomplex + NIR, G5: intratumoral injection FerH cluster + NIR. Data are presented as mean ± SD. (n = 5). Statistical significance was calculated via one-way ANOVA with Tukey's multiple comparisons. (*p < 0.05, **p < 0.01, ***p < 0. 001).

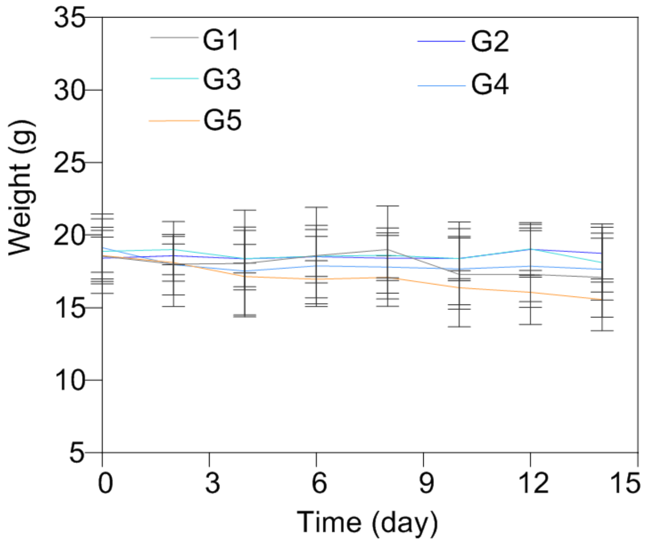

Figure S25. Bodyweight of mice after being treated with different treatments for 14 days. G1: intratumoral injection PBS, G2: intratumoral injection FerH nanocomplex, G3: intravenous injection FerH nanoaggregates + NIR, G4: intratumoral injection FerH nanocomplex + NIR, G5: intratumoral injection FerH nanoaggregates + NIR. Data are presented as mean ± SD. (n = 5).

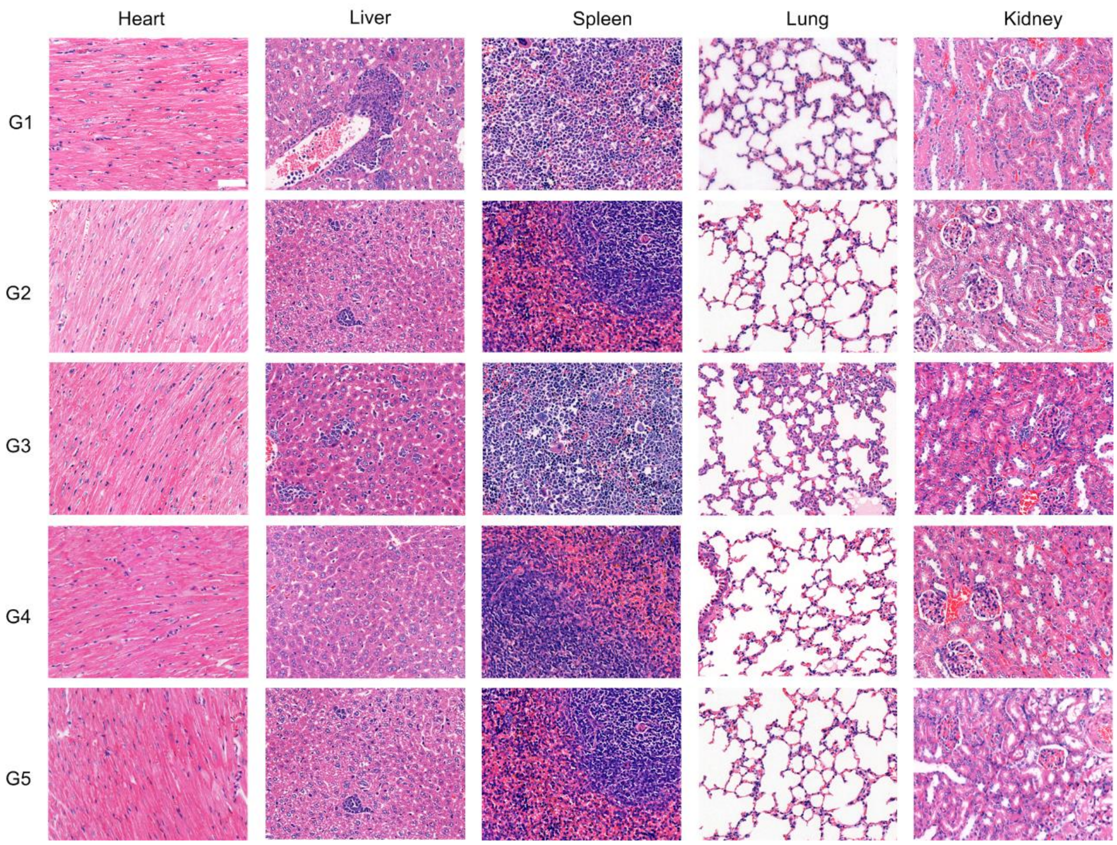

Figure S26. Safety evaluation of FerH induced photothermal therapy on tissues and organs. H&E staining of the mice's major organs (heart, liver, spleen, lung, kidney) to evaluate the histological differences after different treatments. Scale bar, 50 µm.

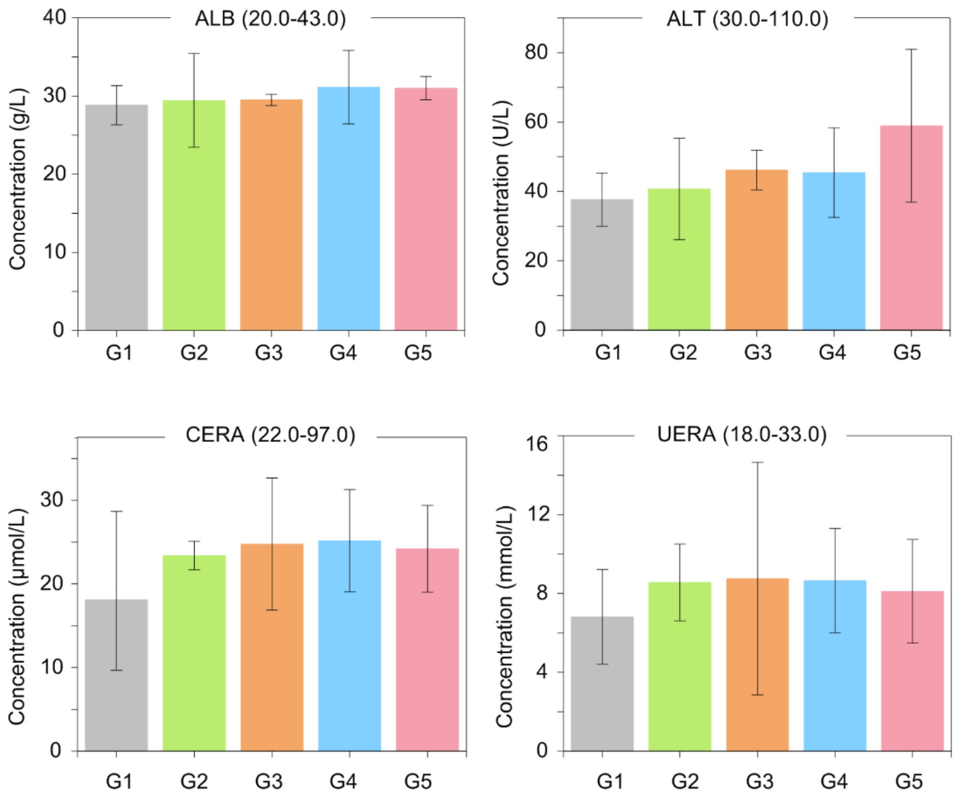

Figure S27. Blood biochemistry analysis of mice after different treatments. The biomarkers include ALB, ALT, CREA, and UREA. Data are presented as mean ± SD. (n = 3).

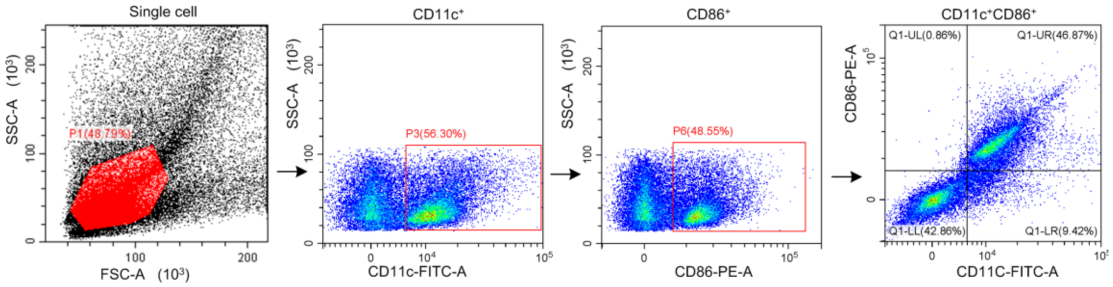

Figure S28. Gating strategies of DC maturation analysis in tumor draining lymph nodes.

Reference:

1. Wang L, Bao J, Wang L, Zhang F, Li Y. One-pot synthesis and bioapplication of amine-functionalized magnetite nanoparticles and hollow nanospheres. Chem - A Eur J. 2006; 12: 6341–7.
